# Shape modeling of longitudinal medical images: from diffeomorphic metric mapping to deep learning

**DOI:** 10.3389/frai.2025.1671099

**Published:** 2025-10-30

**Authors:** Edwin Tay, Nazli Tümer, Amir A. Zadpoor

**Affiliations:** Department of Biomechanical Engineering, Faculty of Mechanical Engineering, Delft University of Technology (TU Delft), Delft, Netherlands

**Keywords:** deep learning, shape modeling, spatiotemporal, medical imaging, diffeomorphisms, longitudinal data

## Abstract

Living biological tissue is a complex system, constantly growing and changing in response to external and internal stimuli. These processes lead to remarkable and intricate changes in shape. Modeling and understanding both natural and pathological (or abnormal) changes in the shape of anatomical structures is highly relevant, with applications in diagnostic, prognostic, and therapeutic healthcare. Nevertheless, modeling the longitudinal shape change of biological tissue is a non-trivial task due to its inherent nonlinear nature. In this review, we highlight several existing methodologies and tools for modeling longitudinal shape change (i.e., spatiotemporal shape modeling). These methods range from diffeomorphic metric mapping to deep-learning based approaches (e.g., autoencoders, generative networks, recurrent neural networks, etc.). We discuss the synergistic combinations of existing technologies and potential directions for future research, underscoring key deficiencies in the current research landscape.

## 1 Introduction

“*Form follows function,”* although originally a perennial maxim coined by architect Louis Sullivan in reference to pragmatic architectural design, it has been adopted by the biomedical engineering community in reference to nature and its adaptability (Sullivan, 1896; Russell et al., 2000). This phrase is often used in reference to natural materials, which have optimized their shape and structures over millennia of evolution and adapted to their specialized tasks (Wegst et al., 2014). While studies have investigated both *form* and its effect on *function* (Libonati and Buehler, 2017; Wang Y. et al., 2020), how it *follows* remains nebulous. In particular, the way in which the shapes of anatomical structures change over time has long interested the biomedical engineering community, dating back to and even predating the seminal works of Darwin (2009) and Thompson (1992). Modeling and predicting the evolving characteristics of anatomical geometry is relevant, with applications for clinical diagnoses, prognoses, and interventional treatments. Therefore, uncovering the underlying processes governing shape change of anatomical structures over time remains a highly relevant and developing domain of research.

**Figure 1:**
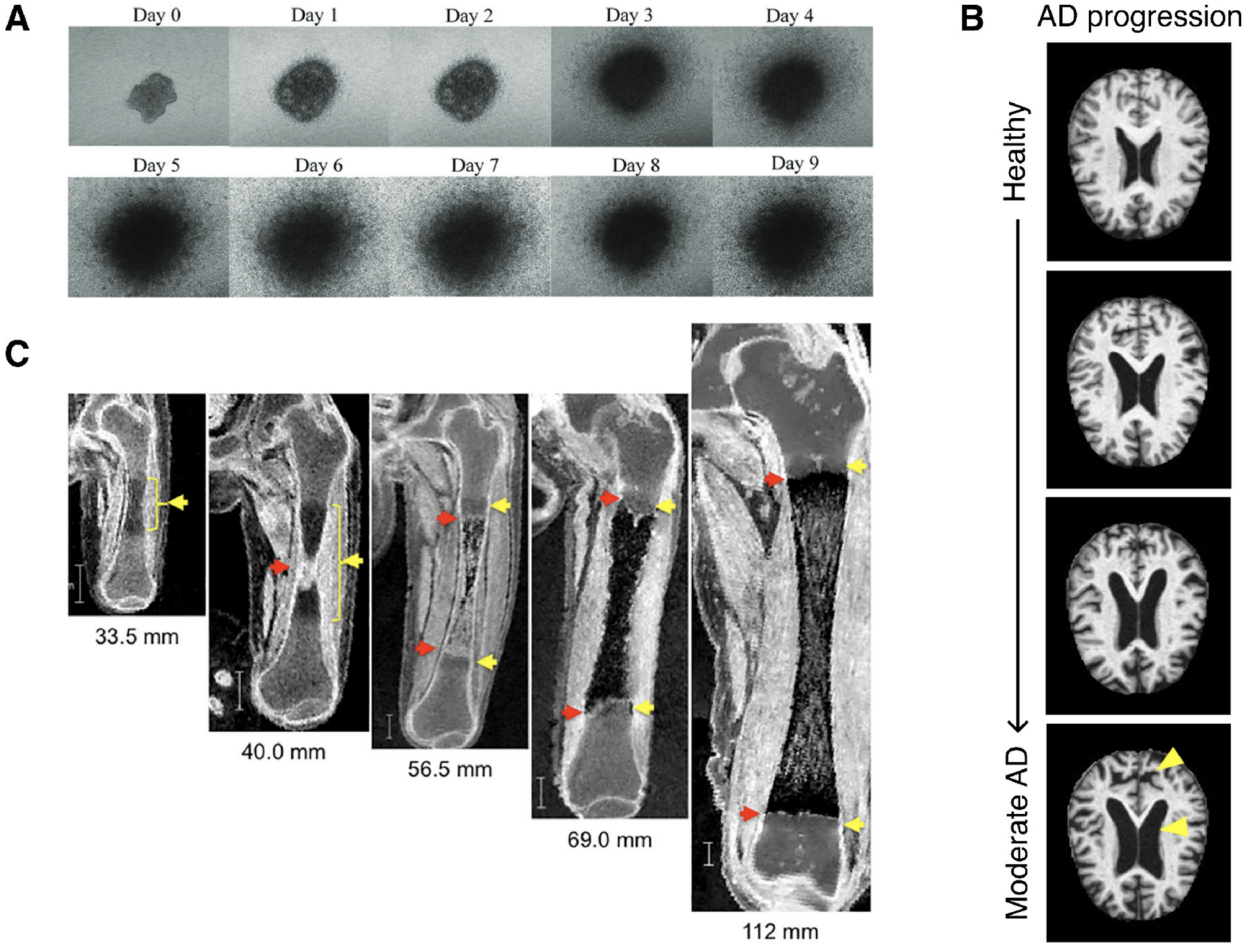
**(A)** Longitudinal phase contrast imaging of 3D cell cultured cervical cancer spheroids (Muniandy et al., 2021)*. **(B)** Neurodegradation of brain structure with progression of AD, from healthy to moderate AD (top to bottom). Adapted from Pasnoori et al. (2024)*. **(C)** Longitudinal MRI imaging of the morphogenesis of a femur during the embryonic and fetal periods. Figure adapted from Suzuki et al. (2019)*. *Images obtained from referenced sources and licensed under CC BY 4.0 (https://creativecommons.org/licenses/by/4.0/).

Longitudinal changes in the shapes of anatomical structures are relevant in a myriad of clinical applications, especially for early diagnosis and disease prognosis (Figure 1). Developmental bone growth, for example, is a highly complex process wherein deficiencies or deviations from nominal standards could result in long-term health ramifications (Parfitt et al., 2000; Weaver and Fuchs, 2014). Some examples of such disorders include but are not limited to developmental hip dysplasia, osteogenesis imperfecta, scoliosis, and clubfoot (Morcuende and Weinstein, 2003). Early diagnosis could enable non-surgical treatments. Therefore, accurate ways of quantifying normal development and identifying abnormal variations is paramount (Semler et al., 2019; Marzin and Cormier-Daire, 2020; Newsome et al., 2016). Another example is Alzheimer's disease (AD), one of the most common age-related neurodegenerative diseases (Scheltens et al., 2021). Commonly used techniques for early diagnosis of AD, such as neuropsychological tests, are unreliable and cerebrospinal-fluid biomarker measurements are intrusive and costly (Alberdi et al., 2016). In contrast, novel techniques examining structural brain changes from MRI can diagnose AD early and pre-symptomatically, while also informing future prognoses (Mueller et al., 2005; Pegueroles et al., 2016; Blinkouskaya and Weickenmeier, 2021). Yet another example is tumor growth, wherein growth rates and tumor sizes inform cancer severity and prognoses (Clark, 1991; Morikawa et al., 2011; Kuroishi et al., 1990). Thus, developing spatiotemporal growth models of tumors has been a long-standing field of research, ranging from early simplified deterministic 1-D models to more complex probabilistic simulations (Adam, 1986; Jiang et al., 2005; Rejniak and Anderson, 2010; Gerlee, 2013; Benzekry et al., 2014). While not an exhaustive list, these examples demonstrate the wide-ranging applications and clinical relevance of developing robust spatiotemporal shape modeling tools and methodologies.

Early spatiotemporal shape modeling can be linked to morphometrics, wherein researchers attempted to analyze biological shape variation using statistical methods. Generally speaking, researchers analyzed variations of common anatomical landmarks across a population (Slice, 2007). These analyses examined variations in the coordinates of landmarks themselves, distances or relative angles between them, or metrics calculated from a combination thereof (Rohlf, 1990; Slice, 2005; Bookstein, 1982). In the femur, for example, measurements such as the whole femur length, diaphyseal length, subtrochanteric anteroposterior and mediolateral diameters, anteroposterior physeal angles, alpha angle, and vertical diameter of the femoral head are some of the measurements used to characterize femoral anatomy (Toogood et al., 2008; Wescott, 2005; Rissech et al., 2008). These methods, however, are time-consuming and landmark placement can be unreliable, inconsistent, and fail to capture holistic spatial arrangement. Developments in computer vision and mathematical modeling tools have led to the development of computational anatomy (CA) (Miller et al., 1997; Grenander and Miller, 1998; Miller, 2004). Therein, the concept of shape manifolds and diffeomorphic transformations became central in describing anatomical shape variability over time. These tools have developed greatly in recent years. Furthermore, with the advent of deep learning (DL), novel methodologies have surfaced. Outlining available methodologies along with their strengths, weaknesses, and potential synergies is, thus, required.

In this review, we seek to highlight and discuss alternative techniques, methodologies, and tools used to model the changing shape of anatomical structures over time. For simplicity and due to the varied terminologies found in the literature, we use the terms spatiotemporal shape modeling and longitudinal shape models interchangeably. We also focus mainly on the techniques and tools themselves as opposed to their clinical applications. However, we highlight applications as necessary to enhance the descriptive value of the presented concepts. We also neglect exhaustive discussions on these methods' mathematical background and derivations, and instead refer the readers where necessary. We do, however, provide some further mathematical detail surrounding the discussed techniques in the accompanying supplementary document which is organized in parallel to the main text. Here, we refer to *shape* in both a geometric sense (i.e., a set of points in *n*-dimensional Euclidean space (ℝ^*n*^) with defined connections) and also in the intuitive sense of a visual boundary defining an object of interest within an image. This is important as both definitions play a role in the differing techniques we explore. A relatively similar review was carried out by Harie et al. (2023), however they explicitly focused on growth modeling and mainly discussed DL-based generative networks. In contrast, this review focuses on shape change over time in general, thus encompassing both growth and alternative biological processes (e.g., degeneration). Furthermore, this review does not focus exclusively on DL-based methods and also covers alternatives. This review begins with a discussion on diffeomorphisms and large deformation diffeomorphic metric mapping (LDDMM) framework, the most common early framework for spatiotemporal shape modeling. Then, we discuss deep learning-based tools, focusing on autoencoders, generative adversarial networks, recurrent neural networks, transformers, and diffusion models. Finally, we discuss the strengths and drawbacks of each tool generally, highlighting similarities and potential synergies. We also speculate on potential future outlooks and directions for research into spatiotemporal shape modeling.

## 2 Large deformation diffeomorphic metric mapping

Of the many ways to describe variations in shapes in biology, a longstanding idea was first proposed by Thompson in his influential work “*On Growth and Form”* in 1917 (Thompson, 1992). Therein, he argued that variations in the shapes of biological organisms can be best described by geometrical transformations. This pioneering theory formed the basis for CA decades later with the development of computer vision and mathematical tools. In essence, CA assumes that individual shapes are described as diffeomorphic transformations of an underlying reference shape. As, in principle, an infinite number of diffeomorphisms can act on a reference shape, sets of diffeomorphisms can then be considered as an infinite dimensional manifold (Marsland and Sommer, 2020). Accordingly, all the possible variations of a given shape can be represented within these manifolds, which are termed as “shape spaces” (Kendall, 1984; Monteiro et al., 2000; Rohlf, 2000). These manifolds can then be enriched with Riemannian metrics which enable quantitative comparison of these shapes and further mathematical operations (Younes, 2010; Miller et al., 2002; Miller, 2004). This constitutes the basis for large deformation diffeomorphic metric mapping (LDDMM) framework (Glaunès et al., 2008; Durrleman et al., 2014). Wherein, variations of anatomical shape in a population are described *via* diffeomorphisms acting on an underlying reference template. These diffeomorphisms then make up the shape space, an infinite dimensional Riemannian manifold describing all possible variations of a shape in a population. The LDDMM framework can then be extended further for longitudinal shape modeling as we will discuss.

### 2.1 Geodesics

**Figure 2:**
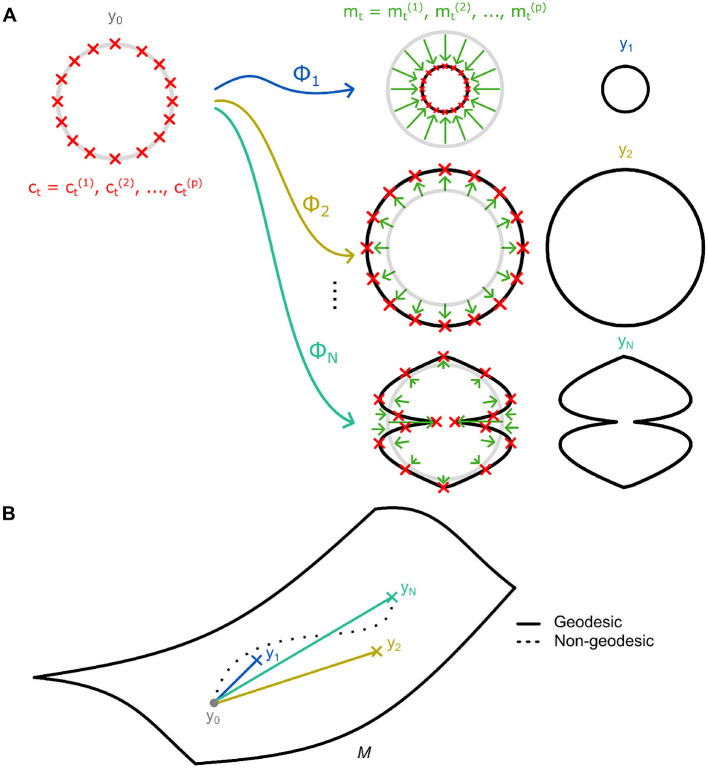
**(A)** An illustration of diffeomorphisms [ϕ_1_, …, *ϕ*_*N*_] acting on a baseline reference shape *y*_0_ to transform it to a shape within a dataset [*y*_1_, …, *y*_*N*_]. A diffeomorphism constitutes *p* momentum vectors *m*_*t*_ acting on a similar number of control points *c*_*t*_. **(B)** Diffeomorphisms within the LDDMM framework lie on a Riemannian manifold *M*. The shortest paths (i.e., geodesics) connecting the reference shape and other shapes are used to describe the transformation and are determined based on minimal deformational energy.

For an initial reference shape *y*_0_ and target shape *y*_1_, a diffeomorphism *ϕ*_1_ exists which can be applied to transform the former to the latter (Figure 2A). Following the convention of Bône et al. (2020a), we denote this as *y*_1_ = *ϕ*_1_⋆*y*_0_. These diffeomorphisms *ϕ*_*t*_ can be difficult to obtain and describe, especially for complex shapes and deformations. Nevertheless, Miller et al. (2006) demonstrated that these complex deformations could be succinctly described by utilizing the principle of conservation of momentum (Vaillant et al., 2004). Specifically, by discretizing them as Gaussian convolutions *g* of *p* momentum vectors $m_{t}=m_{t}^{\left(1\right)},...,m_{t}^{\left(p\right)}\in {\mathbb{R}}^{d}$ acting over a set of corresponding control points $c_{t}=c_{t}^{\left(1\right)},...,c_{t}^{\left(p\right)}\in {\mathbb{R}}^{d}$ (Figure 2A). Nevertheless, solutions for *ϕ*_*t*_ are non-unique due to the infinite-dimensional nature of the underlying shape space manifold. Thus, the geodesic, that is the diffeomorphism requiring the least amount of deformational energy (Figure 2B), is utilized (Miller et al., 2002; Durrleman et al., 2014). Of note is that the geodesics' control points and momenta are also fully determined by their initial values (see Supplementary material S1.1 for further detail). This is particularly notable as, then, the system of initial momenta and control point locations *S*_0_ = {*c*_0_, *m*_0_}, fully parametrize the entire flow of diffeomorphisms.

### 2.2 Geodesic regression

In representing each shape in a longitudinal dataset as a diffeomorphism of a template shape, the challenge remains in establishing the relationships between each shape. This is particularly important as deriving any underlying relationships between different shapes and independent variables (i.e., time) is essential for spatiotemporal shape modeling. Acquiring these relationships *via* standard regression techniques, for instance, is non-trivial due to the non-Euclidean structure of the Riemannian manifolds of diffeomorphisms. Nonetheless, Fletcher proposed an extension of standard linear regression to be applicable in a manifold-based setting, termed geodesic regression (Fletcher, 2011; Thomas Fletcher, 2012). Their technique was then developed further for a variety of applications, but the developments of Fishbaugh et al. (2013) and Fishbaugh et al. (2017) for use in longitudinal shape modeling are of particular interest for our purposes.

**Figure 3:**
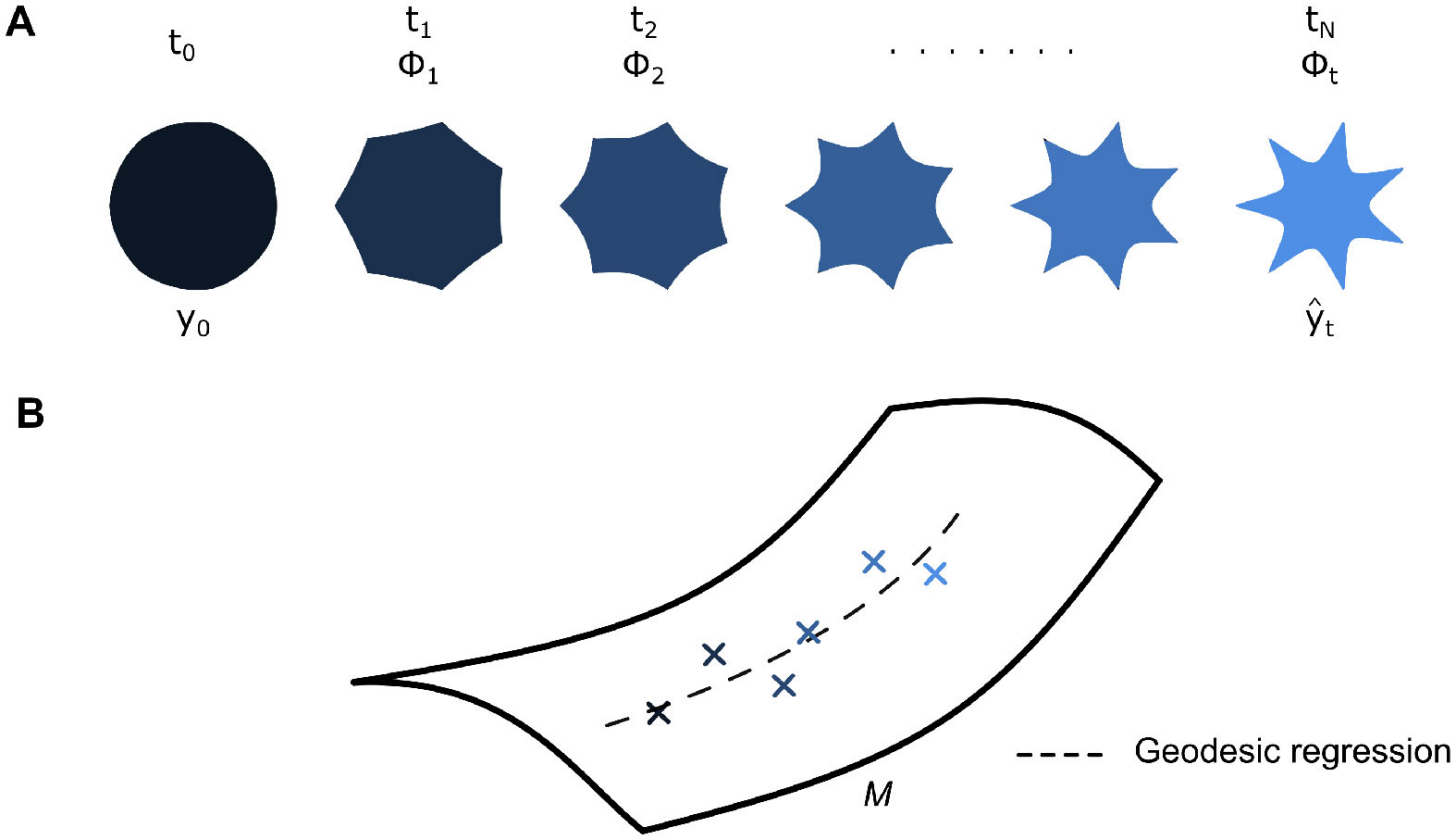
**(A)** Each shape in a longitudinal dataset of *N* shapes spanning [*t*_0_, *t*_*N*_] can be described with a corresponding diffeomorphism at time *t*, *ϕ*_*t*_, acting on reference shape *y*_0_. These diffeomorphisms are obtained from the estimation of an underlying group-average geodesic. Thus, the action of *ϕ*_*t*_ on *y*_0_ leads to an estimate for the corresponding shape $\hat{y_{t}}$ **(B)** Each diffeomorphism lies on a Riemannian manifold *M*, and an underlying group-average geodesic, which describes the trajectory of diffeomorphisms, can be estimated *via* geodesic regression.

In detail, for a longitudinal dataset of shapes with *N* number of observations in the time range [*t*_0_, *t*_*N*_], shape change over time is taken as a baseline shape *y*_0_ being continuously deformed at each time point *t* by a corresponding diffeomorphism *ϕ*_*t*_ (Figure 3A). In principle, *ϕ*_*t*_ should lead to the baseline shape morphing to completely match the observed shape *y*_*t*_ = *ϕ*_*t*_⋆*y*_0_. However, in this context of estimating a holistic group-average geodesic (Figure 3B), we note that a diffeomorphism instead leads to an estimation of the observed shape at time *t* instead $\left(\hat{y_{t}}=\phi_{t}⋆y_{0}\right)$. A regression criterion can then be expressed as follows (Equation 1) (Fishbaugh et al., 2013, 2017).


(1) $$\begin{array}{l} E\left(y_{0},S_{0}\right)=\overset{N}{\underset{i=1}{\sum }}\frac{1}{2\gamma^{2}}||\left(\phi_{t_{i}}⋆y_{0}\right)-y_{t_{i}}|{|}^{2}+L\left(S_{0}\right), \end{array}$$

where, *L*(*S*_0_) represents a regularity term for the time-varying deformation, determined by the kinetic energy of the control points at *S*_0_ (Supplementary Equation S3). γ^2^ represents a term used to balance the importance between the data and regularity terms. Thus, given a dataset of longitudinal shapes, during the minimization of Equation 1 the baseline shape *y*_0_, initial control point locations *c*_0_, and initial momenta *m*_0_ are the parameters which are estimated. This general form of geodesic shape regression was then developed further to incorporate both shape and image data based on a weighted joint optimization routine (Fishbaugh et al., 2014). Their multimodal approach demonstrated improved performances as opposed to exclusive shape or image approaches. Nevertheless, optimization schemes to solve for the underlying geodesic regressions are computationally expensive, especially for large-scale image datasets. Recently, Ding et al. (2019) have also proposed methods to enhance their speed and effectiveness using DL. They demonstrated that the use of encoder-decoder networks with GPU acceleration could increase computation speeds, enabling the scaling up of studies toward larger datasets encompassing more subjects or longer timescales. Developments notwithstanding, these methods were limited to single subject regressions; geodesic regression up to this point has mainly captured spatiotemporal variability of only single subjects, thus these methods were extended further to capture populations and their intervariabilities.

### 2.3 Hierarchical models

**Figure 4:**
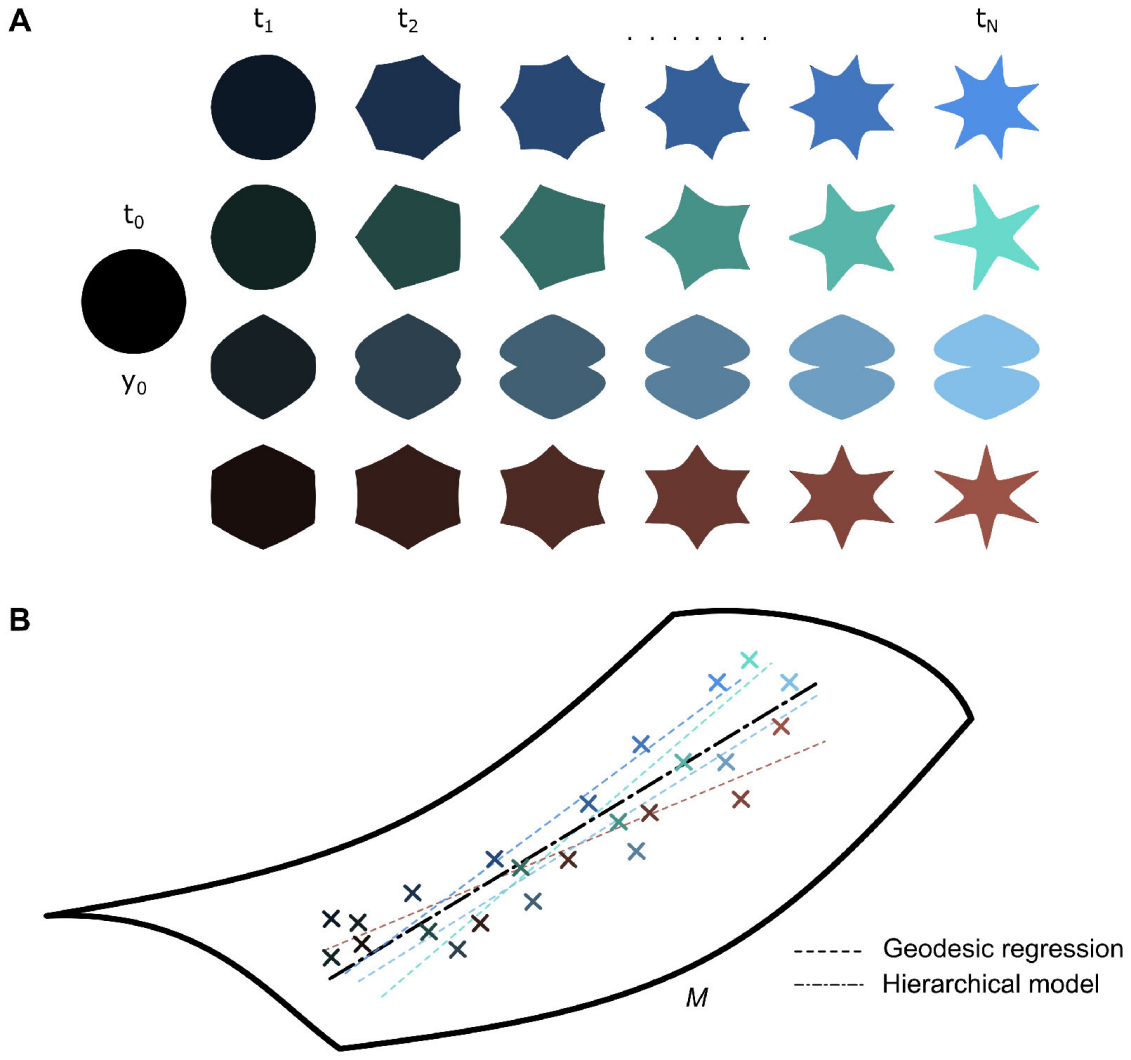
**(A)** A dataset of various shapes spanning [*t*_0_, *t*_*N*_] can be described as diffeomorphic transformations of an underlying baseline template shape *y*_0_. **(B)** Individual shape trajectories can be modeled by individual geodesic regressions, which can be used to estimate a group-average geodesic or vice-versa (*i.e*., group-average geodesic used to estimate individual trajectories).

While geodesic regression can describe an object's longitudinal trajectory over time, it is insufficient to describe the longitudinal characteristics of multiple objects in a large dataset (Figure 4A). Thus, the LDDMM framework was further extended toward hierarchical models. Early work done by Muralidharan and Fletcher (2012) could estimate an underlying groupwise mean geodesic based on individual geodesics (Figure 4B). They did this with a least squares estimation of the underlying mean geodesic, using Sasaki metrics to compare individual trends. This was developed further by Singh et al. (2015) as a generalization of hierarchical linear models to a manifold-based setting. Schiratti et al. (2017) took a slightly different modeling approach, wherein they first found the underlying group average spatiotemporal trajectory and represented individual trajectories within the dataset as space and time transformations of this group-average. This approach offers more flexibility as, unlike the former approach, it is not heavily dependent on initial time point choice, easing time reparametrization. Bone et al. (2018) and Bône et al. (2020a) developed this approach further for shape data within the LDDMM framework specifically.

Briefly, the hierarchical generative longitudinal models of Bone et al. (2018) and Bône et al. (2020a) rely on exp-parallelization (${\text{ExpP}}_{\gamma }^{v_{i}}$) and a time warp function (ψ_*i*_) to account for individual spatial and temporal differences respectively (Equation 2). Exp-parallelization essentially offers a tool to define parallel curves on a manifold whilst retaining the underlying structure (Schiratti et al., 2015, 2017). This enables us to define individual trajectories traversing the manifold as a variation of a group average. A time warp, on the other hand, accounts for the temporal characteristics of each individual's trajectory (*i.e*., onset time, and rate of progression).


(2) $$\begin{array}{l} {\text{ExpP}}_{\gamma }^{v_{i}}\;\left[\psi_{i}\left(t_{i,j}\right)\right]⋆y_{0}\overset{\text{iid}}{\sim }N_{\epsilon }\left(y_{i,j},\sigma_{\epsilon }^{2}\right)\\ \text{where }|\psi_{i}:t\to \alpha_{i}\cdot \left(t-\tau_{i}\right)+t_{0}\\ \;|v_{i}=\text{Conv}\left(c_{0},m_{i}\right),\;m_{i}=A_{0,m_{0}^{⊥}}\cdot s_{i} \end{array}$$

In turn, each component of the model (Equation 2) is as follows. A prediction for shape observation *j* of subject *i*, *y*_*i, j*_ is modeled as a noisy estimate with variance $\sigma_{\epsilon }^{2}$. *y*_*i, j*_ itself is predicted as a diffeomorphic transformation of a baseline reference shape *y*_0_ transformed by an underlying group average geodesic γ space-shifted by exp-parallelization to match an individual's trajectory *v*_*i*_. The time warp function ψ_*i*_ accounts for temporal characteristics, where α_*i*_ denotes progression rate, τ_*i*_ is onset time, and *t*_0_ is the reference time. *v*_*i*_ accounts for the individuals' spatial variability and, in essence, is Supplementary Equation S2 with some additional constraints. Namely, the momenta *m*_*i*_ are obtained from a mixing matrix $A_{0,m_{0}^{⊥}}$ and *q* source parameters $s_{i}=s_{i}^{\left(1\right)},...,s_{i}^{q}$. Note that the mixing matrix serves to project the source parameters into the higher dimensional momentum space (see Bone et al., 2018; Bône et al., 2020a for further detail). The parameters to be estimated which define individual trajectories are modeled as independent samples from normal distributions:


(3) $$\begin{array}{l} \alpha_{i}\overset{\text{iid}}{\sim }N_{\left[0,+\infty \right]}\left(1,\sigma_{\alpha }^{2}\right)\\ \tau_{i}\overset{\text{iid}}{\sim }N\left(t_{0},\sigma_{\tau }^{2}\right)\\ s_{i}\overset{\text{iid}}{\sim }N\left(0,1\right) \end{array}$$

Taken together, a mixed effects model can be defined for the gathered parameters. Fixed effects, which account for parameters affecting the trajectories of all the subjects, can be denoted as θ = (θ_1_, θ_2_). Where, θ_1_ = (*t*_0_, σ_τ_, σ_α_, σ_ϵ_) and θ_2_ = (*y*_0_, *c*_0_, *m*_0_, *A*_0_). The random effects *z*_*i*_ account for variations for each subject, where *z*_*i*_ = (α_*i*_, τ_*i*_, *s*_*i*_). This nonlinear multi-parameter optimization task is computationally complex and expensive and relies on a multi-step *calibration, personalization*, and *simulation* scheme detailed further in Bône et al. (2020a). In brief, it utilizes a novel Monte Carlo Markov Chains-Stochastic Approximation Expectation Maximization-Gradient Descent (MCMC-SAEM-GD) algorithm detailed further in the reference.

### 2.4 Applications and further works

Overall, the use of hierarchical models provides us with a structured framework to characterize longitudinal data, both on an individual and group level. The use of a group-average trajectory enables us to quantify the variation of an individual's progression from a normative scenario (Kim et al., 2017). This also has the potential for prognostic benefits. For example, Cury et al. (2016) could detect shape changes in the thalamus of patients suffering from dementia 10 years prior to clinical symptoms by comparing healthy and diseased spatiotemporal trajectories. Bône et al. (2017) have demonstrated the use of exp-parallelization and time reparametrization to transport a population average trajectory onto new subjects. Thus, they demonstrated that population-average normative trajectories can be leveraged to predict trends in shape change or disease progression for new, unseen subjects. Similarly, Koval et al. (2018) implemented a manifold-based hierarchical model but in the context of graph networks. Specifically, they derived a population-based estimate for cortical atrophy dynamics and demonstrated the capability to characterize patient-specific atrophy dynamics. They further extended this work to account for multimodal data such as biomarker levels and cognitive impairment scores to develop a comprehensive spatiotemporal atlas of Alzheimer's disease (Koval et al., 2021). This method of integrating the use of biomarkers (i.e., genetic and clinical factors) alongside imaging has gained traction and not only demonstrates soundness in and of itself (Dalca et al., 2015) but also has the potential to enhance the predictive capacity of existing frameworks with multimodality. Couronne et al. (2019) further demonstrated the efficacy of multimodal models in the context of Parkinson's disease prognosis. Utilizing both imaging and neurophysiological test score data, they demonstrated the robustness and efficacy of multimodality to improve predictive performance.

Nevertheless, the hierarchical model framework is still being developed further to refine its modeling efficacy and integrate newer technological developments. As opposed to modeling correlations along a manifold as quasi-linear in the manner of geodesics, Hanik et al. (2022) proposed to utilize generalized Bézier curves to model nonlinear relationships with the rationale that many biological processes are nonlinear (e.g., cardiac motion). Their initial work demonstrated the potential for extending this principle further and potentially decomposing longitudinal trends (i.e., disease progression) into different components of a nonlinear curve, enabling more granular analyses. Hong et al. (2019) also investigated the effects of subject-specific characteristics by including multivariate intercept models in their formulation of a hierarchical geodesic model. Debavelaere et al. (2020) developed a methodology to investigate datasets with heterogeneous populations (i.e., a dataset with diverging longitudinal dynamics). They developed an unsupervised algorithm that is able to detect clusters of subgroups within a dataset and differentiate their trajectories, accounting for diverging or converging trajectories from a population normal. Gaudfernau et al. (2023) also extended the LDDMM framework via multiscale representations of images and demonstrated improved results on fetal brain growth estimation, a comparatively more difficult task. Furthermore, the advent of DL has led to augmentations of the LDDMM framework due to its increased computational efficiency of processing large datasets (Yang et al., 2023; Ben Amor et al., 2023). Bône et al. (2019) demonstrated the use of autoencoders to learn an atlas and class of diffeomorphisms that describe a dataset of shapes and meshes. They further extended their work to also account for the texture (i.e., appearance) of images (Bône et al., 2020b). Pathan and Hong (2018) also demonstrated the potential of using DL to learn vector momenta utilized in the LDDMM framework. Other novel developments include the utilization of implicit neural representations (INRs) (Sitzmann et al., 2020). Dummer et al. (2023) demonstrated the potential of using INRs to extend the LDDMM framework toward increased robustness and resolution independence.

To surmise, the LDDMM framework is a powerful tool for representing and modeling a dataset of shapes. Assuming an underlying template shape, the LDDMM framework represents individual shapes as diffeomorphic transformations of this template. These diffeomorphisms lie on an infinite dimensional Riemannian manifold, thus relying on geodesic regression and parallel transport tools to estimate the longitudinal trajectories traversing the underlying data manifold. Hierarchical models can then be utilized to model differing spatiotemporal trajectories of a population, capable of estimating population average spatiotemporal trajectories and also quantifying intra and inter-individual differences. Whilst, in recent years, the proliferation of DL-based techniques has seemingly eclipsed LDDMM-based techniques, the framework is continuously developing. In fact, many of the developments seek to utilize DL tools to accelerate the framework and increase its efficacy. LDDMM methods are readily available in several software packages and applications such as Deformetrica (Bône et al., 2018), Leaspy (https://leaspy.readthedocs.io/en/stable/), and Morphomatics (Ambellan et al., 2021).

## 3 Deep learning

In medical imaging, DL-based solutions have pushed the state-of-the-art further for a variety of tasks. From image segmentation, disease diagnosis, and prognosis to synthetic image synthesis, DL represents a powerful paradigm for the future of medical image analysis (Shen et al., 2017; Wang T. et al., 2020). In this section, we highlight alternative network architectures that have been utilized for spatiotemporal shape modeling.

### 3.1 Autoencoders

**Figure 5:**
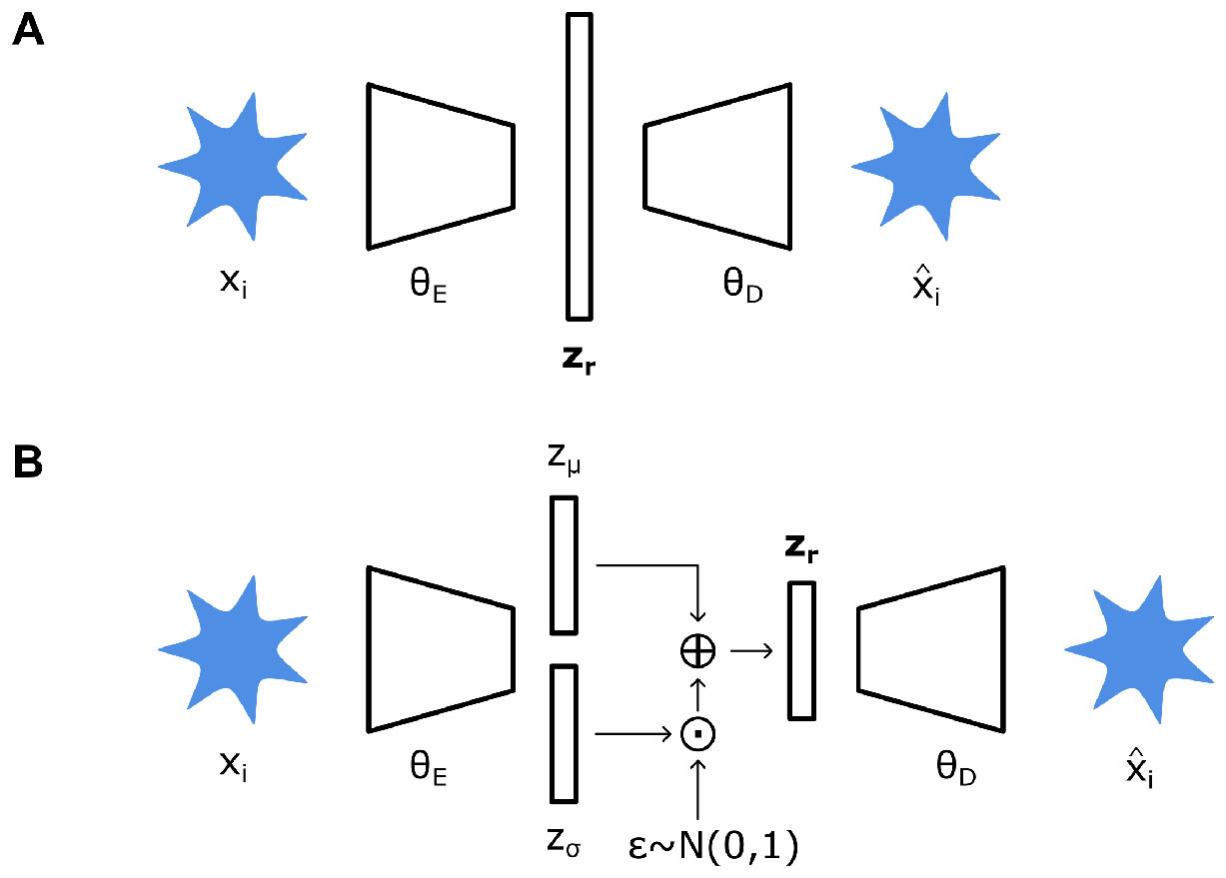
**(A)** Autoencoder structure consisting of an encoder (θ_*E*_) which translates an input image *x*_*i*_ into a vector of latent variables z_r_. A decoder (θ_*D*_) then attempts to reconstruct input data ${\hat{x}}_{i}$ from z_r_. **(B)** A variational autoencoder consists of similar components, however θ_*E*_ maps *x*_*i*_ instead to deterministic parameters *z*_μ_ and *z*_σ_ which describe a probabilistic distribution. These are then used to obtain z_r_ and similarly decoded.

Autoencoders (AEs) are a neural network (NN) architecture consisting of an encoder and decoder module (Figure 5A). This architecture, in principle, seeks to compress data to a low-dimensional latent space, reducing them to *r* number of latent variables, **z**_**r**_. These latent variables themselves can then be utilized for other tasks as they represent, in essence, a compressed low-dimensional representation of higher-dimensional data. Thus, the weights of the encoder θ_*E*_ and decoder θ_*D*_ modules are learned to accurately de-construct input data down into a latent representation and re-construct them into the original input data, respectively (Lopez Pinaya et al., 2020). The objective when training an AE is then to minimize the loss function $L^{rec}$, which takes the form of a dissimilarity function or reconstruction loss, to find θ_*E*_ and θ_*D*_ (Equation 4). Details on the loss function and structuring of regularization can be found in the Supplementary material S2.1.


(4) $$\begin{array}{c} \min L\left(\theta_{E},\theta_{D}\right)=\underset{\theta_{E},\theta_{D}}{\min}\overset{N}{\underset{i=1}{\sum }}L^{rec}\left(x_{i},\hat{x_{i}}\right)\\ \text{where }\hat{x_{i}}=\theta_{D}\left(\theta_{E}\left(x_{i}\right)\right) \end{array}$$

A variation of AEs is variational autoencoders (VAEs) which are similar but treat encoding and decoding in a probabilistic manner (Figure 5B) (Rezende et al., 2014). Instead of directly mapping input data to latent variables, VAEs map input data to probabilistic distributions of their corresponding latent variables. Briefly, θ_*E*_ maps input data to deterministic parameters, mean *z*_μ_(*x*) and standard deviation *z*_σ_(*x*), which describe an underlying probabilistic distribution (usually Gaussian) of the latent space. These deterministic parameters are then injected with stochasticity sampled from a fixed normal distribution, where ⊙ denotes a Hadamard product (Equation 5). This configuration is necessary to preserve the stochasticity within the latent space while enabling gradient-based backpropagation during training (Ehrhardt and Wilms, 2022). In turn, the loss function (Equation 4) is now modified to consider both reconstruction quality and regularity of the latent space (Equation 6). The latter is usually represented by a Kullback-Leibler divergence, detailed elsewhere (Ehrhardt and Wilms, 2022). Overall, a probabilistic treatment of latent variables and the spaces they inhabit leads to more structured, compact, and continuous latent spaces. This, in turn, leads to a smoother sampling of latent variables for generative processes and representation learning in general.


(5) $$\begin{array}{l} z=z_{\mu }\left(x\right)+z_{\sigma }\left(x\right)⊙\epsilon \text{ with }\epsilon \sim N\left(0,1\right) \end{array}$$

(6) $$\begin{array}{l} L^{VAE}=L^{rec}+L^{KL} \end{array}$$

AEs, VAEs, and variations thereof have many applications in generative frameworks and tasks involving reduced dimension representations of high dimensional data such as images. The strengths of these architectures in modeling complex data within low-dimensional representations could lend themselves well to capturing the complex nonlinearities inherent in longitudinal datasets. Latent variables and the spaces they inhabit have been utilized as parameters to be fit to existing models. Sauty and Durrleman (2022) utilized a VAE to learn latent variables representing images within a longitudinal dataset. These latent variables are then fitted to a linear longitudinal mixed-effects progression model similar to those of the LDDMM framework. Chadebec and Allassonnière (2023) utilized normalizing flows to model latent variables representing spatiotemporal data, thus imposing temporal structure onto the latent space. Kapoor et al. (2025) presented MRExtrap, a framework wherein they utilize linear models to model latent variables extracted from a regularized AE. Their framework successfully estimated longitudinal trajectories via a progression rate variable, from a single scan, based on population and subject-specific priors which can be updated dynamically with new data. Nevertheless, as latent variables lack any specific underlying physical meaning, developments in techniques to identify what these variables represent have also been made. Mouches et al. (2021) utilizes an invertible latent space disentanglement module within an autoencoder framework to determine latent variables that affect age-related changes. Isolated age-related latent variables can then be varied, with age-unrelated components kept constant, to simulate the aging of a particular individual. Following a similar vein, Zhao et al. (2021) utilized a cosine-based loss function to disentangle brain age from image representation. They did so with a self-supervised learning methodology, optimizing the correspondence between the “directionality” of latent variables in the latent space and physical developmental trajectories. As opposed to fitting models to latent variables themselves, structuring the latent space during training via conditional priors or regularization is a common and effective technique. He et al. (2025) developed a conditional VAE architecture capable of predicting follow-up MRI scans of the human brain. Ong et al. (2024) incorporated the use of linear mixed models as conditional priors on the latent space of VAEs. Chen *et al*. demonstrated the use of orthogonality mixed-effects constraints to structure the latent space of an autoencoder. Their method could robustly identify both global and local longitudinal trajectories, with enhanced classification outcomes (Chen et al., 2025).

Overall, AEs and VAEs represent powerful tools for reducing high-dimensional data into a low-dimensional latent space, efficiently encapsulating longitudinal data into compressed latent variables. Nevertheless, latent variables and their spaces are solely reduced dimension representations of the original input data (Ehrhardt and Wilms, 2022); latent variables have no underlying physical meaning. For example, the distribution of latent variables has been shown to be affected by training parameters, demonstrating their capricious nature (Lapenda et al., 2020). Thus, latent variables cannot be considered spatiotemporal variables. However, rational structuring and regularization to ensure that latent spaces are enriched with physical meaning can lead to better outcomes. Another point of concern is that both AEs and VAEs generally treat latent spaces and variables in a Euclidean manner, when in fact research has shown that a manifold-based approaches may be more prudent (Connor et al., 2021). These problems remain active fields of research, with solutions such as regularization and explicitly structuring latent spaces deterministically being continually developed (Tschannen et al., 2018; Ghosh et al., 2020). Nonetheless, existing works for spatiotemporal shape modeling demonstrated the potential applicability for autoencoders and learned latent variables to model longitudinal trajectories.

### 3.2 Generative adversarial networks

**Figure 6:**
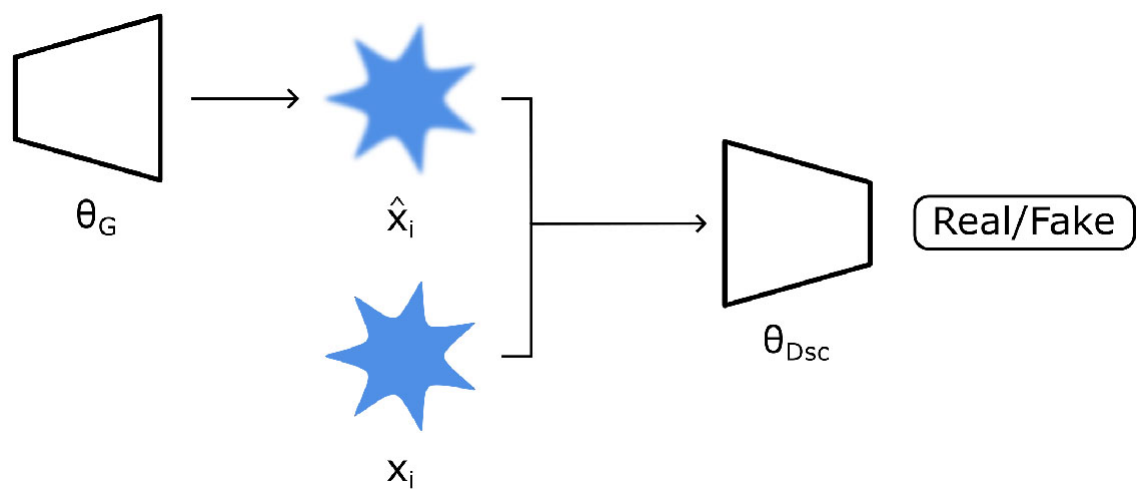
A generative adversarial network (GAN) architecture consists of a generator (θ_*G*_) which creates synthetic data (${\hat{x}}_{i}$) resembling real data (*x*_*i*_). A discriminator (θ_*Dsc*_) then attempts to differentiate real vs. “fake” synthetic data. Both θ_*G*_ and θ_*Dsc*_ are jointly trained so that the former generates increasingly realistic images while the latter is able to discriminate real *vs*. fake data better.

Generative Adversarial Networks (GANs) are neural network architectures first proposed by Goodfellow et al. (2014). In principle, they consist of generator θ_*G*_ and discriminator θ_*Dsc*_ networks being trained simultaneously (Figure 6). Therein, the former is trained to create new synthetic images, whilst the latter is trained to detect if an image is real or fake. In detail, θ_*G*_ maps random input variables ν (sampled from a prior distribution *p*(ν)) to the data space in an attempt to generate data ${\hat{x}}_{G}$ resembling data from a real dataset *x*_*r*_. In turn, both types of data are fed into θ_*Dsc*_, whereby θ_*Dsc*_ is trained to determine if data is real or fake. The objective function used to train both networks simultaneously is then a minimax problem (Equation 7).


(7) $$\begin{array}{l} \underset{\theta_{G}}{\min}\underset{\theta_{Dsc}}{\max}V\left(\theta_{G},\theta_{Dsc}\right)=E_{x\sim p_{data}\left(x_{r}\right)}\left[\log D\left(x\right)\right]\\ \;+E_{z\sim p\left(\nu \right)}\left[\log\left(1-D\left(G\left(\nu \right)\right)\right)\right] \end{array}$$

This architecture is very powerful, as the adversarial training configuration leads to the generator module being capable of generating realistic synthetic images that are indistinguishable from real data (Wang K. et al., 2017; Gui et al., 2023). Trained generators are then useful for many applications. In the context of medical imaging, examples include image synthesis, segmentation, and classification, among others (Yi et al., 2019). Many variants exist, and more are continually being developed, for which the reader is referred to other papers for further details (Jabbar et al., 2021). In the context of spatiotemporal shape modeling, GANs represent a powerful tool. Similar to previously discussed AEs and VAEs, their generative capacity can potentially be utilized to capture the underlying spatiotemporal trajectories.

Elazab et al. (2020) used a stack of 3D GANs to predict brain tumor growth. Specifically, with an input image and physiological feature maps, a generator predicted a brain scan at the proceeding time point whose accuracy was evaluated by a discriminator. Their results outperformed contemporary methods but relied on stacking and training consecutive GANs, which is computationally inefficient. Alternatively, Zhang et al. (2017) utilized GANs to uncover the underlying data manifold of longitudinal progression for face aging. They first encode images to latent vectors, which are concatenated with age-related feature vectors and then mapped onto a manifold. Discriminators ensure regularized latent vector generation and image realism of the generators. Based on this, Ravi et al. (2019) developed a 2D framework to model age-related brain degeneration in the context of Alzheimer's diagnosis. They incorporated further voxel-based and region-level constraints which acted as biological constraints to model Alzheimer's progression, leading to improved prognoses. They developed this work further to examine 3D MRIs for a more holistic view of the brain (Ravi et al., 2022). Utilizing a 3D training consistency mechanism and a super-resolution module led to a full 4D model of brain aging without a loss in anatomical detail. Following the same principle of temporal embedding within a latent space, Schön et al. (2023) similarly implemented a GAN-based network for embedding temporal directionality in generators. Alternative GAN architectures have also been investigated. Wasserstein GANs (WGANs) utilize Wasserstein distances as a loss function as opposed to regularly used Jensen-Shannon divergence (Arjovsky et al., 2017). This architecture leads to more stable training outcomes and was utilized by Wegmayr et al. (2019) as a recursive generator model to predict time steps in brain aging. Combined with a classifier network, they present a framework for both predicting aged brain images and Alzheimer's prognosis, outperforming standard methods. In StyleGAN and derivatives thereof, the principle of style transfer and additional, intermediate latent spaces is utilized to improve generator architectures and disentangle latent space components and their effects on synthesized images (Karras et al., 2019, 2020; Fetty et al., 2020). Han et al. (2022) developed a framework for image-based osteoarthritis prognosis using StyleGAN as the generative architecture. This enabled them to construct the underlying manifold of longitudinal knee aging, and furthermore, they demonstrated that their model outperforms human radiologists in early diagnosis of osteoarthritis. Similarly, Gadewar et al. (2023b) and Gadewar et al. (2023a) utilized StarGAN-v2, a similar style-based generator architecture, to predict aging in structural MRIs of the brain.

In short, GAN-based architectures and adversarial training represent powerful tools for spatiotemporal shape modeling. In particular, discriminators support the structuring and regularization processes so that the latent space of generator modules is physically meaningful, similar to previously discussed regularized AEs. While GANs have their own challenges in terms of training stability, mode collapse, convergence, and image fidelity, continual developments in training schemes, architectures, and loss functions have led to continuous improvements (Yi et al., 2019; Gui et al., 2023; Saxena and Cao, 2021). Generators with well-defined and structured latent spaces, and rational generative processes enable us to predict growth trajectories. Said structuring of latent spaces is facilitated by discriminators and loss functions, which allow us to ensure smooth latent spaces that are temporally consistent and valid. In essence, in helping structure latent spaces, discriminators implicitly define the underlying manifold of spatiotemporal shape progression. Similar to previously discussed AEs, this structuring process ensures that latent spaces and variables therein can be endowed with meaningful physical characteristics.

### 3.3 Recurrent neural networks

**Figure 7:**
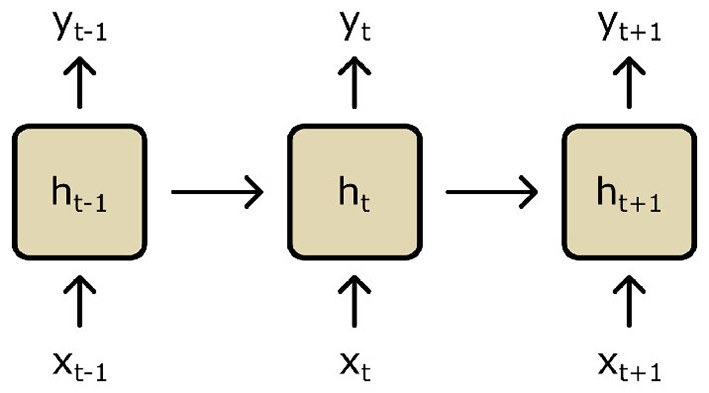
A recurrent neural network (RNN) is trained along a sequence of time points *t*. Based on input (*x*_*t*_) and output data (*y*_*t*_), a hidden state (*h*_*t*_) is continuously updated using context units.

Recurrent neural networks (RNNs) are a type of NN that are used to model sequential data such as a time series (Lipton et al., 2015; Salehinejad et al., 2018; Staudemeyer and Morris, 2019). They do so by considering data along a whole sequence's trajectory during training and inference; RNNs are designed explicitly with features that connect and consider data inputs across longitudinal sequences by maintaining memory (i.e., a hidden internal state *h*_*t*_ which is continually updated at each time point *t*). Early RNNs utilized simple “context units,” which are units independently connected to nodes in the hidden layer of an NN (Figure 7) (Elman, 1990). These context units are then updated along steps in a data sequence *via* activation functions as the RNN is trained along a sequence. These simple context units were then developed to more complex long short-term memory (LSTM) cells to address practicalities surrounding network training (further details in Supplementary material S2.2).

Thus far, LSTMs have been used for natural language processing or other tasks examining relatively low-dimensional data. In the context of images and CNNs, LSTMs have been adapted for image inputs in the form of the convolutional LSTM (ConvLSTM) (Shi et al., 2015). ConvLSTM is able to capture temporal information and dependencies in a sequence of images while ensuring that spatial information is preserved during encoding. This has led to its use and marked effectiveness in video prediction tasks (Lotter et al., 2016; Lu et al., 2017).

In the context of longitudinal medical imaging, RNNs have improved the outcomes of segmentation (Gao Y. et al., 2018) and disease stage classification tasks (Santeramo et al., 2018; Gao L. et al., 2018; Cui and Liu, 2019; Ouyang et al., 2021; Ding et al., 2023). In explicitly modeling shape change using ConvLSTMs and its derivatives, however, RNNs have seen comparatively less uptake potentially due to the significantly high GPU memory requirements (Ma et al., 2022). Some studies nevertheless utilize RNNs as components within larger frameworks to avoid this obstacle. For example, Pathan and Hong used LSTMs to predict the vector momentum sequences to deform a longitudinal baseline image in an LDDMM framework (Pathan and Hong, 2018). This approach leverages the effectiveness of the LDDMM framework to predict changes over time without loss of detail and the computational efficiency of DL. Louis et al. (2019) utilized RNNs to encode longitudinal trajectories into a latent space. These encoded trajectories are then decoded to construct the manifold and the Riemannian metrics lying on this manifold. Ma M. et al. (2023) utilized ConvLSTMs alongside a transformer in a “growth prediction module” to predict tumor growth. They demonstrated that utilizing both components in a unified module leads to better-predicted growth morphologies. Zhang et al. (2020) extended the ConvLSTM framework with the goal of modeling spatiotemporal sequences (ST-ConvLSTM). Their ST-ConvLSTM units learn both temporal and spatial dependencies in a sequence; for a 3D image slice, ST-ConvLSTM learns both the changes over time for that slice and accounts for the adjacent slices.

To surmise, RNNs represent a powerful network architecture for capturing temporal dependencies within a longitudinal dataset. Nevertheless, the issue of high GPU memory requirements for imaging data persists. This particular requirement precludes the use of RNNs for longitudinal shape modeling. Nevertheless, Ma et al. (2022) and Ma Z. et al. (2023) sought to address this by developing multi-scale RNN frameworks, which demonstrably improve performance with much lower GPU memory costs. Chen et al. (2024) demonstrated the use of signed distance function-based representations with ConvLSTMs to predict longitudinal changes in the shape of vestibular schwannoma. They demonstrated a proof of concept for using signed distance functions, which could address issues of large memory requirements of conventional ConvLSTMs operating directly on images. All in all, developments in using LSTMs for medical imaging datasets are relatively recent and have yet to be fully investigated in the context of longitudinal medical image shape modeling.

### 3.4 Transformers

**Figure 8:**
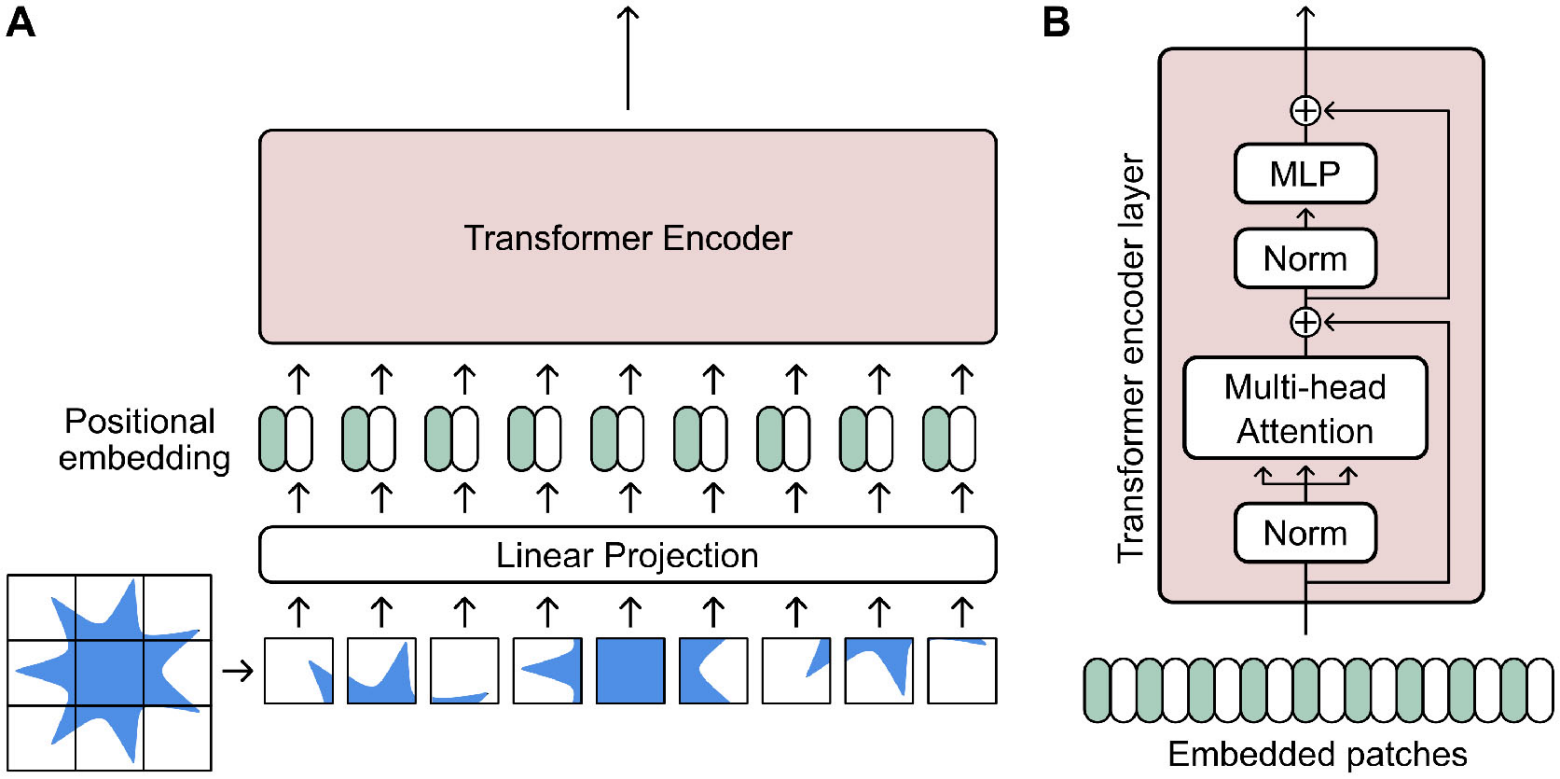
**(A)** A Visual Transformer (ViT) architecture tokenizes an input image by first delineating it into smaller patches. Each patch is then linearly projected and embedded alongside its positional data before being fed into a transformer encoder. **(B)** A transformed encoder layer takes the embedded image patches as tokens and uses them within a multi-head attention-based encoder layer. Figure inspired by existing work of Dosovitskiy et al. (2020).

Transformers are a relatively recent development in DL. Originally designed for natural language processing (NLP) tasks (Vaswani et al., 2017), they utilize a novel attention mechanism based on saliency, which can capture long-range dependencies in data sequences. The architecture was later developed further specifically for image data with the Vision Transformer (ViT) framework (Dosovitskiy et al., 2020). In any case, transformer networks rely firstly on tokenization of input data (Figure 8) (Shamshad et al., 2023; Islam et al., 2024; Torralba and Isola, 2024). This process essentially entails subdividing input data into “tokens,” wherein each token is passed to an attention module where they can be used to calculate an attention score (further details in Supplementary material S2.3)

These tokenized representations and attention modules are then integrated into various NN architectures and can be configured for many applications, especially in medical image analysis (Azad et al., 2024). In particular, the capability to capture long-range dependencies and focus on salient features across long input sequences could potentially be applicable for predicting shape changes over time sequences.

In the context of longitudinal shape modeling, the use of transformers are still relatively unexplored. Sarasua et al. (2021) was one of the first to apply transformers to model longitudinal shape trajectories. They forecasted the change in the shape of meshes of the left hippocampus in an encoder-decoder-style architecture utilizing a bidirectional transformer encoder. They extended this work further by explicitly embedding Alzheimer's cognitive impairment scores and utilizing pre-trained transformers (Sarasua et al., 2022). The latter method revolved around freezing most layers of a pre-trained transformer and fine-tuning it on a selected task to decrease the number of trainable parameters (Lu et al., 2021). The former method of embedding cognitive scores was also similarly utilized by Xia et al. (2021) to synthesize longitudinal brain images. With an input baseline brain image, their transformer architecture embeds a health state and age progression to synthesize changes over time. To improve the quality of their predicted progressions, they trained their networks in an adversarial manner with additional loss functions to preserve subject identity. Wang et al. (2022) developed a comprehensive transformer-based framework to predict tumor growth. Their so-called static-dynamic framework utilizes a transformer-based module to first encode and enhance high-level features of detected tumors. Then, a transformer-based growth estimation module is employed to predict growth based on the aforementioned extracted features.

Nevertheless, applications of transformers for longitudinal shape modeling is still in its relative infancy. Advances in transformer architectures, such as incorporating multi-scale convolutions for enhanced time-series prediction, could potentially be applied to imaging data as well (Wang and Guan, 2023). However, there are a number of caveats to the enhanced performance of transformer-based networks (Li et al., 2023). Firstly, the nature of the transformer architecture leads to lower degrees of inductive bias, necessitating larger amounts of training data for better performance. This could potentially be addressed with pre-training as demonstrated by Lu et al. (2021), but nevertheless remains a consideration. Furthermore, training transformer architectures is computationally expensive, requiring significant computing resources, especially if applied to 3D volumetric medical imaging. In fact, a relatively high number of studies in the field are focused on reducing this computational burden (Xia and Wang, 2023). This heightened computational resources required thus present a barrier, prohibiting widespread development and applications to new data. Early studies have already demonstrated promising results, and transformer architectures could present a future avenue for spatiotemporal shape modeling.

### 3.5 Diffusion models

**Figure 9:**
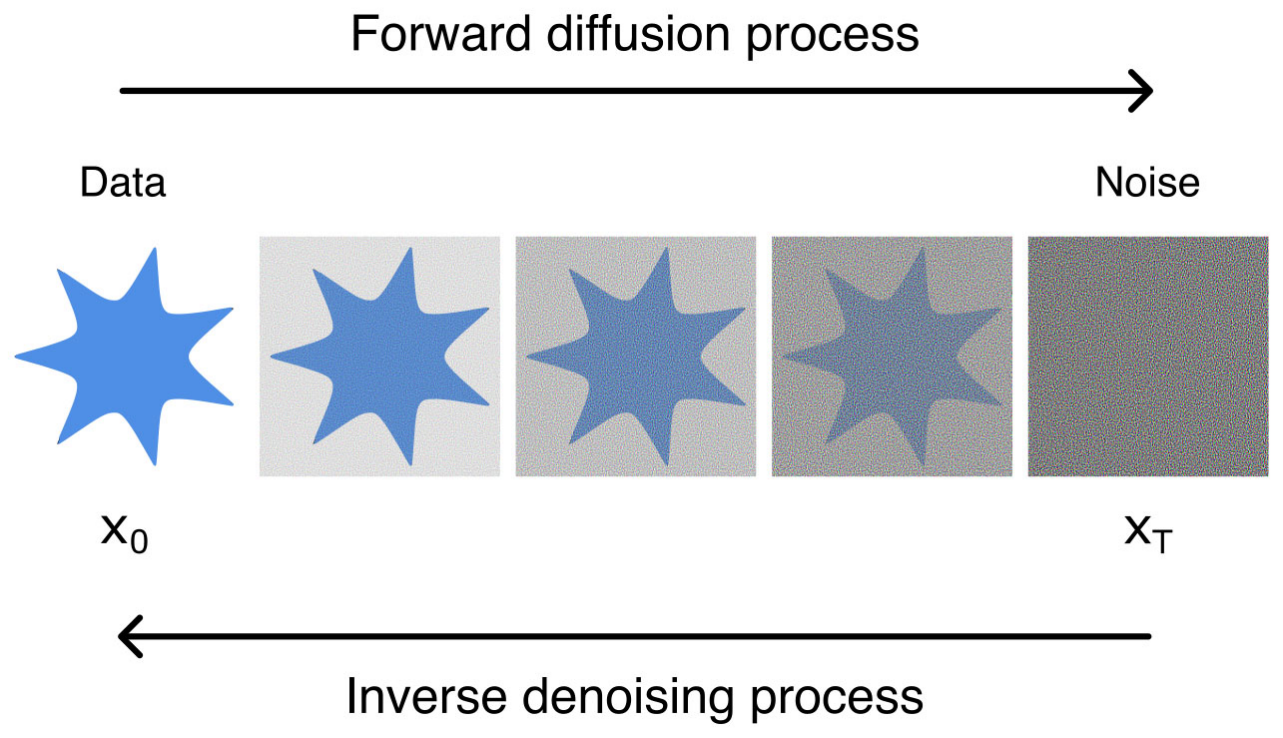
Illustration of forward and inverse diffusion process for diffusion models.

Diffusion models (DMs) are a type of generative DL architecture similar to aforementioned GANs and AEs. In contrast, however, DMs function on the principle of noise addition and removal (Fuest et al., 2024; Croitoru et al., 2023; Kazerouni et al., 2023); DMs consist of forward and inverse processes, wherein noise is added onto input data in successive steps, and the resulting noise is reversed to reform the input data (Ho et al., 2020) (Figure 9).

These processes are Markovian in nature, and the forward process is generally handcrafted (i.e., manually chosen or optimized for). The inverse process, however, is what is learned by the network. In detail, the process is a Markov chain which starts from a data distribution *q*(*x*_0_) and a sequence of *T* steps corrupting it to N(0,1), a Gaussian distribution, with Markov diffusion kernels *q*(*x*|*x*_*t*−1_) (Equation 8).


(8) $$\begin{array}{l} q\left(x_{1},x_{2},...,x_{T}|x_{0}\right)=\overset{T}{\underset{t=1}{\prod }}q\left(x_{t}|x_{t-1}\right)\\ \;q\left(x_{t}|x_{t-1}\right)=N\left(x_{t};\sqrt{1-\beta_{t}}x_{t-1},\beta_{t}I\right) \end{array}$$

Where β_*t*_ is the variance of noise and *I* is the identity matrix. The reverse denoising process is what is learned by the model, that is inverting the diffusion process and turning the latent noise variable *p*_θ_(*x*_*t*_) back into the data distribution *p*_θ_(*x*_0_) parameterized by θ (Equation 9).


(9) $$\begin{array}{l} p\left(x_{0},x_{1},...,x_{T-1}|x_{T}\right)=\overset{T}{\underset{t=1}{\prod }}p_{\theta }\left(x_{t-1}|x_{t}\right)\\ \;p_{\theta }\left(x_{t-1}|x_{t}\right)=N\left(x_{t-1};\mu_{0}\left(x_{t},t\right),\sigma_{0}^{2}\left(x_{t},t\right)I\right)\\ \;p\left(x_{t}\right)=N\left(x_{T};0,I\right) \end{array}$$

Where, $\sigma_{0}^{2}\left(x_{t},t\right)$ is variance at step *t* and μ_0_(*x*_*t*_, *t*) is the mean of the Gaussian distribution. Thus, from a randomly sampled noise vector, novel samples can be generated (for further mathematical detail, readers are referred to Fuest et al., 2024; Croitoru et al., 2023; Kazerouni et al., 2023).

DMs have led to state-of-the-art high resolution visual generative networks (Rombach et al., 2021; Dhariwal and Nichol, 2021), and existing works have demonstrated the potential for use in a variety of spatiotemporal modeling tasks (Yang et al., 2024; Rühling Cachay et al., 2023). Yoon et al. (2023) demonstrated the use of a sequence-aware diffusion model (SADM) to generate longitudinal medical images. Their framework utilized a sequence-aware transformer as the conditional module for a diffusion model, demonstrating effective data generation capabilities for longitudinal 3D medical imaging sequences, even with missing data. Litrico et al. (2025) utilized patient metadata and age gaps to condition their diffusion model, demonstrating effective results. Lozupone et al. (2025) took an alternative approach, wherein they applied the diffusion process to a compressed latent representation of their images as opposed to the images themselves. This approach enables computationally efficient processing of 3D medical imaging and demonstrated impressive results on disease classification and temporal trajectory prediction. Puglisi et al. (2025) followed a similar approach in their Brain Latent Progresion (BrLP) framework. Alongside training a DM to operate on latent variables, they further condition it with additional patient metadata and anatomical measures. Operating within their wider framework, they demonstrate state-of-the-art results in trajectory prediction.

While a promising avenue of development, DMs are still a developing field with many deficiencies to be addressed. Namely, a main issue is with computational efficiency (Croitoru et al., 2023; Guo et al., 2023). The multi-step noising and denoising processes takes more computational time and resources compared to other generative networks, precluding potential real-time diagnostic applications. Training these networks also requires considerable computational resources which potentially surpass the requirements of alternative networks. Whilst developments in improving efficiency of DMs have been made, it is still a growing field of interest (Shen et al., 2025). Furthermore, to ensure the validity of generated data, auxiliary networks such as ControlNet and variants thereof have been developed to enhance the tractability of DMs' generative processes (Zhang et al., 2023; Yang et al., 2025). Integration of DMs into wider frameworks such as those incorporating attention-based mechanisms has also demonstrated to be effective, and further developments could also be promising (Wu and Gong, 2024).

### 3.6 Limitations of deep learning approaches

Although the presented DL techniques have led to great strides forward in the state-of-the-art of spatiotemporal shape modeling, several challenges persist. These challenges hinder the widespread adoption of DL both in general and medical image analysis specifically. Thus, practitioners should be aware that DL cannot be simply considered a panacea for their tasks.

Firstly, DL models are inherently “black boxes” (Castelvecchi, 2016): while their outputs might be accurate, valid, and valuable according to many objective metrics, the inscrutability of how these outputs arise is a predominant concern. The opaque nature of how trained models arrive at their solutions engender doubts regarding trustworthiness due to the lack of explainability (Li et al., 2022; Xu and Yang, 2025). Especially for medical applications, interpretability and understanding is paramount. In modeling the progression of diseases for example, both for observational and prognostic applications, developing an understanding of the phenomena being modeled can be more important than the final model output itself (Young et al., 2024). Thus, efforts have been made to increase the explainability of these models and increase levels of trust with clinical end users (Singh et al., 2020). For example, a popular technique for image classification is via class activation maps (Teng et al., 2022). This visualization technique essentially highlights the specific discriminative image regions which influence final classification output. Many alternative methods exist such as utilizing accompanying language models to provide elaborative textual explanations (i.e., captions) describing visual results (Patrício et al., 2023). Schutte et al. (2021) also demonstrated the use of StyleGAN to generate counterfactual images as an alternative illustrative means of increasing interpretability.

Another concern is the implicit biases embedded into trained DL models. As DL models generally have no underlying physical grounding, their overall performance is entirely dependent on the quality of input training data and susceptible to biases or errors in the data itself (the garbage in, garbage out principle) (Geiger et al., 2021). This is related to the disproportionate prevalence of White, Educated, Industrialized, Rich, and Democratic (WEIRD) datasets in the field of behavioral sciences (Henrich et al., 2010). This is an issue given that while WEIRD populations do not represent the global norm, they are overrepresented in academic research. This is also an underlying problem with DL and available training data. Septiandri *et al*. found that a majority of datasets utilized by researchers at two AI-focused conferences were WEIRD. This could risk under-representing less privileged populations, impeding equal availability of state-of-the-art models or even leading to harmful outcomes (Mihalcea et al., 2025). For example, Puyol-Antón et al. (2021) and Puyol-Antón et al. (2022) demonstrated clear racial biases in segmentation models, attributed to training data composition. Nevertheless, they demonstrated the use of several alternative strategies to address this bias such as training separate population-specific models and ensuring population-balanced training data. Regardless of specific strategies, practitioners should be cognizant of this issue for all DL applications, including spatiotemporal shape modeling, and actively work to address and mitigate these biases.

Domain shift is yet another significant barrier to widespread adoption of DL. Simply put, domain shift refers to dissimilar training and target datasets of a DL model, leading to a lack of generalizability (Guan and Liu, 2022). Especially for medical image analysis, domain shift is an underlying issue and arises from several issues. For example, MRI and X-ray data gathered from different centers examining similar structures can exhibit differences due to differing scanners or acquisition protocols. These differences, while seemingly negligible, greatly degrade downstream performance on trained models (Guo et al., 2024; Pooch et al., 2020). In histopathological data as well, data acquired from different scanners or subject to different pre-processing steps also exhibit degraded downstream performance (Stacke et al., 2019). Nevertheless, addressing this issue (i.e., domain adaptation) remains an active field of research and an underlying consideration to develop robust DL models (Guan and Liu, 2022; Singhal et al., 2023).

Lastly, an unavoidable consideration for many practitioners is the resource requirements for DL. State-of-the-art networks are continually growing in size and complexity, and require an unsustainably increasing amount of compute resources to train (Thompson et al., 2020). Whilst algorithmic improvements to decrease these compute costs for training are being developed, this remains a problem for widespread adoption (Bartoldson et al., 2023). End users of trained models could also encounter high computational costs for model inference, depending on their size and complexity. Higher compute requirements also generally translate to higher monetary costs to access said resources. Similarly, methods are being developed to address efficiency from a monetary as opposed to a compute standpoint (Klemetti et al., 2023). Nevertheless, these requirements could present a simple but significant barrier to resource-limited practitioners seeking to develop or train models further.

## 4 Discussion and conclusions

Several approaches for spatiotemporal shape modeling of anatomical structures were discussed in this review. Rapid developments in the field, especially in recent years, have been fueled by advancements in DL and are set to only continually progress further. Nevertheless, the works found in the existing literature have been mainly focused on incremental developments in methodology or applications of novel new tools. This is in contrast with applying already developed tools to existing or novel clinical challenges. This seeming reluctance of the medical imaging community toward application-based research could stem from a multitude of reasons, but a simple lack of data could be the main factor, as we will discuss shortly. Deficiencies notwithstanding, in this section, we will discuss key concepts of spatiotemporal shape modeling uncovered from our review. We will then outline several key barriers to further research in the field before speculating on future research directions.

### 4.1 Nonlinear shape manifolds

From our review, it is clear that anatomical shape variation is highly nonlinear. This nonlinearity is further compounded by the additional nonlinear dynamics of growth and changing biological structures over time, leading to an intricate and complex outlook. Thus, the best-suited models for spatiotemporal progression are those that lie on non-Euclidean manifolds as they best capture this inherently high dimensional problem. A potential reason for this could be the manifold hypothesis, wherein it is postulated that all high-dimensional data lie on an embedded low-dimensional manifold (Fefferman et al., 2016; Narayanan and Mitter, 2010). The task of spatiotemporal shape modeling can then be reduced to identifying and characterizing these manifolds, either implicitly or explicitly. The LDDMM framework discussed in Section 2, for example, explicitly seeks to uncover spatiotemporal trajectories of diffeomorphisms traversing across a manifold. DL techniques discussed in Section 3 also implicitly benefit from manifolds, as the efficacy of DL techniques has been attributed to their capability to uncover and disentangle underlying the manifolds of complex data (Brahma et al., 2016).

In contrast to manifold-based techniques, several works do exist that have attempted to extend linear (PCA-based) statistical shape models toward spatiotemporal shape models. These, however, fall short when compared to LDDMM and DL-based solutions as they effectively only serve to compare differences across and interpolate between time points as opposed to true longitudinal forecasting (Hamarneh and Gustavsson, 2004; Kasahara et al., 2018; Binte Alam et al., 2020; Saito et al., 2019). Due to their reliance on landmarks, these methods do not effectively work if anatomies significantly change over time, as is the case, especially in early development. Furthermore, they are incapable of separating groupwise vs. individual developmental trends, nor are they capable of effective data imputation (Adams et al., 2023). Therefore, while these methods might be effective for comparing shape variation across time points, they are not as effective for shape trajectory forecasting as manifold-based methods.

Comparatively, manifold-based techniques are more effective as the longitudinal trajectories traversing the shape space yield an effective description of shape variation over time. LDDMM techniques offer a structured framework to describe shape variation, and furthermore, the geodesic trajectories themselves are clinically relevant as they offer prognostic and diagnostic utility. When utilized within a hierarchical model that incorporates many trajectories for a population average, new trajectories can be estimated for unseen data, which could offer prognostic significance. Furthermore, trajectories can be compared using relational transport operators to diagnose if a trajectory is irregular compared to population averages. Similarly, DL methods mostly operate directly on medical imaging data with convolutional networks. This allows us a way to extract hidden features from images which could also influence spatiotemporal trajectories, otherwise lost during parameterization processes necessary for LDDMM or PCA-based models. In encoding networks especially, the latent space encompassed by these extracted latent variables can be structured to construct a physically meaningful underlying spatiotemporal manifold. The inductive capacity of DL methods with such structured latent spaces is then superior to linear methods, capable of imputing missing data and predicting spatiotemporal trajectories.

### 4.2 Paucity of longitudinal datasets

Another clear deficiency is the lack of large, open-source, and high-quality longitudinal imaging datasets. Existing datasets used in studies are generally small, in-house, cover a short time span, and are limited to very specific clinical conditions (Table 1). This is, of course, understandable as it is extremely difficult to gather longitudinal data. Issues such as participant attrition (Young et al., 2006) and ethical concerns (Tinker et al., 2009) are just two examples of difficulties that hamper the execution of effective studies. An exception to this is the Alzheimer's Disease Neuroimaging Initiative (ADNI) database, which is a large multimodal database of longitudinal biomarker and neuroimaging data tracking the progression of AD (Jack et al., 2008). This dataset is particularly outstanding due to its size and comprehensiveness, leading to many studies covered in this review validating their methods on the ADNI dataset. Nevertheless, this dataset remains unique and standout compared to others. This paucity of longitudinal datasets, especially for medical imaging, impairs the efficacy of both LDDMM and DL techniques covered in this study.

**Table 1:** A summary of several longitudinal medical imaging datasets.

**Name**	**Anatomy**	**Modality**	**Age range**		**Number of subjects**	**Repeated measurements**
			**Youngest**	**Oldest**		
OASIS-2	Brain	MRI	60	96	150	≤ 5
OASIS-3	Brain	MRI	42	95	1,378	≤ 7
HABS-HD	Brain	MRI	40	92	3,838	≤ 3
Harvard aging brain study	Brain	MRI	62	90	>290	≤ 3
ADNI-4	Brain	MRI	55	90	>2,400	≤ 6
UK Biobank	Brain	MRI	44	87	>5,000	≤ 2
	Whole body	DXA			≥5,156	
	Abdomen	MRI			≥113,65	
	Heart	MRI			≥5,100	
MCSA	Brain	MRI	30	89	1,802	Unknown

OASIS, Open Access Series of Imaging Studies; HABS-HD, Health and Aging Brain Study-Health Disparities; ADNI, Alzheimer's disease neuroimaging initiative; MCSA, Mayo clinic study of aging.

DL techniques are notoriously data hungry, with larger dataset sizes contributing significantly toward improved efficacy of networks (Sun et al., 2017; Cho et al., 2015). While techniques such as transfer learning (Alzubaidi et al., 2020) and data augmentation (Mumuni and Mumuni, 2022) seek to ameliorate this issue, it remains pervasive. Conversely, whilst the LDDMM framework is comparatively not as data-hungry, sufficiently sized datasets are also essential. Adequately sized and diverse datasets are vital to ensure that the estimated population average trajectories are reflective of the entire population. Solutions such as GAN-based frameworks discussed in Section 3.2 are shown to be helpful in addressing the issue of data paucity. Therein, generative processes and adversarial training frameworks can increase the generalizability of networks. The latter is particularly useful as the adversarial process assists in regularizing and structuring the latent space, implicitly learning the underlying spatiotemporal manifold. Nonetheless, the lack of datasets presents another issue of validity. In essence, the impressive performance on specific datasets could be a function of the dataset and not the frameworks themselves. Thus, exploring their efficacies on additional anatomical structures and imaging modalities is also prudent. Initiatives to compile multimodal datasets to train and test frameworks in a challenge-like style such as the Medical Segmentation Decathlon (MSD) could be warranted to ensure that future developments in methodology are sufficiently valid (Antonelli et al., 2022).

Nevertheless, longitudinal datasets, be it open-source or in-house, remain scarce. Gathering additional longitudinal data remains the most ideal option, however the aforementioned practical difficulties in data gathering present a significant barrier. In the medium to long term, additional initiatives resembling ADNI could be warranted to gather high-quality, longitudinal, multi-center data for other diseases and disorders benefiting from spatiotemporal shape analyses. Furthermore, these data should be multi-modal, encompassing both imaging and also biomarker data as these have been shown to work synergistically when incorporated into joint frameworks, improving their efficacy. In the meantime, efforts to compile existing data into a large open-source database could be more warranted. This could resemble, for example, the aforementioned MSD. Nonetheless, the impetus to gather and unite such datasets is lacking, especially in the face of general (un)willingness to openly share rare datasets (Tedersoo et al., 2021). Furthermore, medical imaging datasets face strict international data privacy regulations (Lo, 2015; Phillips, 2018). Regulations such as General Data Protection Regulation (GDPR) and the Health Insurance Portability and Accountability Act (HIPAA) are compounded with ethical concerns and additional practicalities such as ensuring patient anonymity (Lo, 2015; Larson et al., 2020; Banja et al., 2021). Overall, these considerations are non-trivial and present significant barriers to nurturing a culture of open science for spatiotemporal shape modeling.

### 4.3 Comparison of methods

This review focused on two main avenues for spatiotemporal shape modeling, including the LDDMM framework and varying DL approaches. Each strategy presents distinct advantages and disadvantages as we will discuss (Table 2).

**Table 2:** Comparison of approaches for spatiotemporal shape modeling.

**Aspect**	**LDDMM approach**	**Deep learning approaches**
Temporal modeling	Explicit via diffeomorphic trajectories and time warping	Implicit through network architecture (RNNs, transformers) or latent space structuring
Data requirements	Moderate—Can work with smaller datasets due to strong priors	High—Generally require large datasets for good performance
Computational complexity	High—Requires iterative optimization for hierarchical models, computationally expensive geodesic calculations	High for training, moderate for inferences; varies by architecture (transformers >RNNs >CNNs)
Scalability	Limited—Struggles with large datasets, optimization becomes prohibitive	Good—Naturally handles large datasets
Interpretability	High—Explicit mathematical framework, geodesics have geometric meaning, trajectories directly interpretable	Low—Black box nature, latent variables lack physical meaning, requires *post-hoc* interpretability methods
Clinical applicability	Strong for trajectory analysis, population vs individual variation assessment	Strong for end-to-end tasks (e.g., prediction) and multimodal data integration

From the computational complexity viewpoint, the state-of-the-art LDDMM approaches we describe can be computationally expensive. Even with parallelized processing utilizing both CPU and GPU, moderately sized data require up to a day of compute time (Bône et al., 2020a). As discussed in Section 3.6, state-of-the-art DL approaches (e.g., transformers, diffusion models, etc.) also require extensive computational resources for training and potentially inference. Nevertheless, as briefly discussed in the same section, decreasing the cost of training and inference is an active field of research and heavily architecture-dependent.

On interpretability, the LDDMM framework is based on a clear underlying mathematical framework. The uncovered underlying longitudinal trajectories generally present directly interpretable and understandable outputs. Especially with regards to the discussed hierarchical models, which explicitly separate population-level and individual-level variations, the overarching findings taken as immediately interpretable by clinical end users. In contrast, DL models are “black boxes” with opaque decision processes as previously discussed in Section 3.6.

For clinical translation, both approaches offer benefits for different applications. The LDDMM framework allows us to elucidate upon the underlying mechanisms of shape change over time by presenting us with a foundation to separate and study both population and individual dynamics mathematically. In contrast, DL demonstrates superior performance for specific tasks such as prediction or imputation via well-regulated generative architectures. Furthermore, DL models can extract hidden features from imaging data which are not specifically accounted for, compared to an explicit mathematical model for example. Nevertheless, as the decision-making process is opaque without additional *post-hoc* techniques to increase interpretability, it remains difficult to directly apply to a clinical setting as previously discussed (Section 3.6). Thus, the clinical translatability of both approaches is heavily dependent on the end-user and their requirements, be it end-to-end tasks (*e.g., classification, segmentation, etc*.) or understanding and characterization.

### 4.4 Future outlook and directions

In this review, we focused mainly on the development of LDDMM and DL-based techniques. We did so because these were considered the most versatile for generalizable spatiotemporal shape modeling. This is opposed to alternative methods seeking to model shape changes of specific anatomical structures over time from a mechanistic standpoint. For example, many early works on spatiotemporal shape modeling of tumors attempted to develop models uncovering the underlying mechanistic cause and effects governing their growth (Jarrett et al., 2018). Similar works also exist focusing on cardiac tissue remodeling (Wang V. Y. et al., 2017) and bone remodeling (Kameo et al., 2020). Whilst these varied mechanistic models are inherently different, they generally revolve around shape change as a consequence of mechanical and biochemical stimuli or a combination thereof. Thus, these models seek to uncover the underlying formulae governing these interactions and their relationships. This is in contrast with the LDDMM framework, which operates solely from a geometric perspective in uncovering the trajectories of diffeomorphic transformations. In other words, the LDDMM framework does not explicitly consider the underlying physical laws governing the biological processes that lead to the resulting shape changes. Therefore, this approach potentially neglects key information that may affect how reflective the LDDMM approach is in said processes and, therefore, its accuracy. Similarly, DL techniques are opaque, often referred to as black boxes (Castelvecchi, 2016). Therein, the model layers, in effect, operate on hidden features uncovered during training processes. These have, in principle, no physical meaning and are not always explainable, engendering issues of trust and validity.

A compromise and potential future direction of research is via physics-informed neural networks (PINNs) (Cuomo et al., 2022). Therein, the strengths of DL to process large datasets are utilized to solve underlying physical equations that describe the physics of a system. PINNs are particularly useful even, for example, to uncover underlying dynamics of systems that were previously obscured under high dimensional nonlinear data (Lagergren et al., 2020). Tajdari et al. (2021) and Tajdari et al. (2022) demonstrated the applicability of PINN principles within frameworks to model the longitudinal progression of adolescent idiopathic scoliosis, outperforming traditional methods. While their works were mainly concerned with the mechanistic effects of loading on spinal outcomes, their efficacy also lends itself to potential benefits for spatiotemporal shape modeling. Nevertheless, PINNs remain an unexplored avenue and warrant further study.

Another developing field is utilizing and exploiting causality in the form of causal deep learning. In essence, causality and structural causal models (SCMs) seek to capture and model the chain of causality and inter-variability of multivariate systems (Peters et al., 2017; Pearl, 2013; Pearl et al., 2016). This is useful as it enables us to interrogate models to obtain counterfactuals (i.e., if *X* was different, what is the effect on *Y*? Or more relevantly, “How would injury *A* affect bone development of pediatric subject *B*?”). For spatiotemporal shape modeling specifically, this could be used to obtain predictions of shape change over time as a counterfactual from existing data. Traditional methods relied on a system of structural equations with computation graphs, but this precluded the use of higher dimensional data such as images. In recent years, several studies have explored extending SCMs toward being supported by DL [i.e., Deep Structural Causal Models (DSCMs)], enabling the use of hidden features identified via DL (Lore et al., 2018; Pawlowski et al., 2020; Berrevoets et al., 2023). Zhou et al. (2023) reviewed the synergistic capabilities of generative models and causality, specifically highlighting the applicability of the latter in enhancing the interpretability of generative processes. Further works such as by Reinhold et al. (2021) demonstrated the capability of DSCMs to generate counterfactual brain MRIs of patients with multiple sclerosis. They were able to manipulate demographic and disease covariates and observed their effects on MRI imaging in a novel proof-of-concept. Rasal et al. (2022) further extended DSCMs toward shape modeling, specifically 3D meshes, demonstrating the extendability and scalability of the principle toward more complex data types. Nevertheless, the field is still in its relative infancy, with further developments and refinements in the DSCM framework potentially leading to enhanced efficacy for longitudinal shape modeling.

In terms of direct clinical translation, spatiotemporal shape modeling is yet to be fully explored. Existing works covered in this review highlighted, for example, capabilities to stratify patient cohorts both temporally and by subtype (Young et al., 2024; Puglisi et al., 2025). This has the potential capability to enhance the efficacy of clinical trials, for example, by enabling more precise targeting of treatments. To our knowledge, use of longitudinal data modeling is still relatively theoretical and whilst research has projected its utility, direct translations to clinical practice remain difficult as is the case for most biomedical research (Finney Rutten et al., 2024). For our specific context of spatiotemporal shape modeling, our proposed application scenarios (Section 1) are also simply hypothetical at this juncture. Nevertheless, the clinical applicability of spatiotemporal shape modeling remains an unexplored yet promising research domain.

In summary, this paper mainly reviewed the LDDMM framework and DL-based techniques for longitudinal shape modeling. Both achieve their remarkable state-of-the-art performance as they function on similar principles of uncovering the underlying nonlinear spatiotemporal data manifold. Whilst promising, the LDDMM framework is computationally expensive and inefficient due to the exhaustive optimization procedure necessary to calculate smooth and invertible diffeomorphisms. It, nevertheless, demonstrates strong capabilities to establish hierarchical models that differentiate individual and population-level temporal trajectories. Conversely, DL-based techniques are powerful but data-hungry and lack underlying physical meaning. Network architectures have been developed to predict shape changes in anatomical structures. Nevertheless, the underlying data manifolds and spatiotemporal trajectories governing these predictions are obscured by the “black box” nature of DL architectures. This affects the interpretability of these predictions, especially if the longitudinal trajectories themselves are important. Nevertheless, the capability of DL architectures to identify hidden features from input images and implicitly map the underlying data manifolds denote their importance for spatiotemporal shape modeling. State-of-the-art developments in DL such as via foundation models, highly sophisticated pre-trained feature extractors which can extract rich representations from data, are also a promising direction of exploration (Ma et al., 2024; van Veldhuizen et al., 2025; Homayounfar et al., 2026). Our review highlights that hybrid techniques that amalgamate both approaches' strengths are more desirable. Furthermore, frameworks incorporating multi-modal data improved generalizability. Thus, further works should not neglect the utility of auxiliary data (e.g., biomarker levels, demographic information, etc.). Many studies discussed in our review utilized multimodal data, in LDDMM, DL, and hybrid frameworks. Thus, multimodality represents a clear path forward for state-of-the-art development (Nakach et al., 2024). Finally, we theorize that utilizing mechanistic models in a manner similar to PINNs or structured causal frameworks could also further improve the predictive capacities of future spatiotemporal shape models.
