# Supplementary material for: Shape modeling of longitudinal medical images: from diffeomorphic metric mapping to deep learning
Source: Front Artif Intell. 2025 Oct 30;8:1671099. doi: 10.3389/frai.2025.1671099 (PMC12611964; doi:10.3389/frai.2025.1671099)
Supplement: Supplementary file 1 [file Data_Sheet_1.pdf]

frontiers

Supplementary Material

1 LARGE DEFORMATION DIFFEOMORPHIC METRIC MAPPING

1.1 Geodesics

For an initial reference shape y_0 and target shape y_1, a diffeomorphism ϕ_1 exists which can be applied to transform the former to the latter. We can denote this as $y_1 = \phi_1 \star y_0$. Within the LDDMM framework, these diffeomorphisms follow the trajectories of a time-dependent vector field $t \to v_t \in C_0^\infty(\mathbb{R}^d, \mathbb{R}^d)$ over [0,1]. Thus, starting from an identity mapping (Id), the change in ϕ_t over time ($\partial_t \phi_t$) is described as a functional composition of v_t and ϕ_t, denoted by $v_t \circ \phi_t$.(Equation S1).

$$\partial_t \phi_t = v_t \circ \phi_t \quad \text{with} \quad \phi_0 = \text{Id}. \tag{S1}$$

These diffeomorphisms can be difficult to obtain and describe, especially for complex shapes and deformations. Nevertheless, Miller *et al.* demonstrated that these complex deformations could be succinctly described by utilizing the principle of conservation of momentum (*i.e.,* vectors of momenta) Miller et al. (2006); Vaillant et al. (2004). Specifically, v_t can be discretized as a Gaussian convolution g of p momentum vectors $m_t = m_t^{(1)}, ..., m_t^{(p)} \in \mathbb{R}^d$ acting over a set of corresponding control points $c_t = c_t^{(1)}, ..., c_t^{(p)} \in \mathbb{R}^d$, which also influence arbitrary points x (Equation S2).

$$v_t : x \in \mathbb{R}^d \to \sum_{k=1}^{p} g[c_t^{(k)}, x] \cdot m_t^{(k)} \in \mathbb{R}^d \tag{S2}$$

In general notation, the Gaussian kernel function is defined as $g : x, x' \in \mathbb{R}^d \to \exp ||x' - x||_{l^2}^2 / \sigma^2$, with kernel width $\sigma > 0$. Solutions for ϕ_t are non-unique due to the infinite-dimensional nature of the underlying shape space manifold. Thus, the geodesic, that is the diffeomorphism requiring the least amount of deformational energy (Equation S3), is utilized Miller et al. (2002); Durrleman et al. (2014).

$$\frac{1}{2}\int_{t=0}^{1} ||v_t||_{G_{c_t}}^2 = \frac{1}{2}\int_{t=0}^{1} m_t^T \cdot G_{c_t} \cdot m_t \tag{S3}$$

, where, G_{c_t} is the $p \times p$ kernel symmetric positive-definite matrix of general term $g[c_t^{(k)}, c_t^{(l)}]$ and $(\cdot)^T$ denotes a matrix transposition. These geodesics' control points and momenta are also fully determined by their initial values and the following Hamiltonian equations (Equation S4). The former observation is particularly notable as, then, the system of initial momenta and control point locations $S_0 = \{c_0, m_0\}$, fully parametrize the entire flow of diffeomorphisms.

$$\dot{c}_t = G_{c_t} \cdot m_t \quad ; \quad \dot{m}_t = -\frac{1}{2}\nabla_{c_t}\{m_t^T \cdot G_{c_t} \cdot m_t\} \tag{S4}$$

, where ∇_{c_t} is the gradient operator with respect to c_t. Thus, a Riemannian manifold D_{c_0}, based on Equations S1, S2, and S4, can be described as follows (Equation S5):

$$D_{c_t} = \{\phi_1 | \partial_t \phi_t = v_t \circ \phi_t, \quad \phi_0 = \mathrm{Id}, \quad v_t = \mathrm{Conv}(c_t, m_t),$$
$$(\dot{c_t}, \dot{m_t}) = \mathrm{Ham}(c_t, m_t), \quad m_0 \in \mathbb{R}^{p \times d}\} \tag{S5}$$

2 DEEP LEARNING

2.1 Autoencoders

A commonly used loss function is the L2 norm (Equation S6), but alternatives exist that could work better for different forms of input data Khare et al. (2022).

$$\mathcal{L}^{rec}(x, \hat{x}) = ||x - \hat{x}||_2^2 \tag{S6}$$

Latent variables z_r in a standard AE configuration are, in principle, unstructured. Thus, sampling new data in generative processes might not lead to valid data as the properties and ranges of z_r values are not known. Appending regularization terms, $\mathcal{L}^{reg}(z_r)$, to the cost function can lead to more structured latent variables and the spaces they inhabit (Equation S7). These modified AE structures are, thus, referred to as regularized autoencoders Ehrhardt and Wilms (2022).

$$\min \mathcal{L}(\theta_E, \theta_D) = \min_{\theta_E, \theta_D} \sum_{i=1}^{N} \mathcal{L}^{rec}(x_i, \hat{x_i}) + \lambda \mathcal{L}^{reg}(z_i)$$
$$\text{where} \quad \hat{x_i} = \theta_D(\theta_E(x_i)) \tag{S7}$$

λ is a balancing term used to adjust the trade-off between latent-space regularity and reconstruction quality. On the other hand, $\mathcal{L}^{reg}(z_r)$, can take many forms and is detailed further elsewhere Ehrhardt and Wilms (2022); Arpit et al. (2016).

2.2 Recurrent Neural Networks (RNNs)

To address practical issues regarding optimization of network parameters (exploding/vanishing gradients during backpropagation) Lipton et al. (2015); Salehinejad et al. (2018); Staudemeyer and Morris (2019), newer RNN architectures utilize complex memory cells instead of traditional nodes Hochreiter and Schmidhuber (1997); Yu et al. (2019); Chung et al. (2014). A prime example is the Long Short-Term Memory (LSTM) cell. These types of cells utilize 'gates', components that determine when and how to modify the memory of the cell (Figure S1C). In detail, the three main gates of a standard LSTM cell are the forget, input, and output gates. The forget gate utilizes the output of the previous time point y_{t-1} and the current input x_t with a sigmoidal activation function to determine data in h_t should be 'forgotten' (Equation S8). Where W and b denote the weights and biases of the threshold units, respectively.

$$f_t = \sigma(W_f[y_{t-1}, x_t] + b_f) \tag{S8}$$

Similarly, for the input gate, a sigmoidal activation function determines which values are to be updated (Equation S9), and a $\tanh$ activation function creates new values to be added to the internal state (Equation S10).

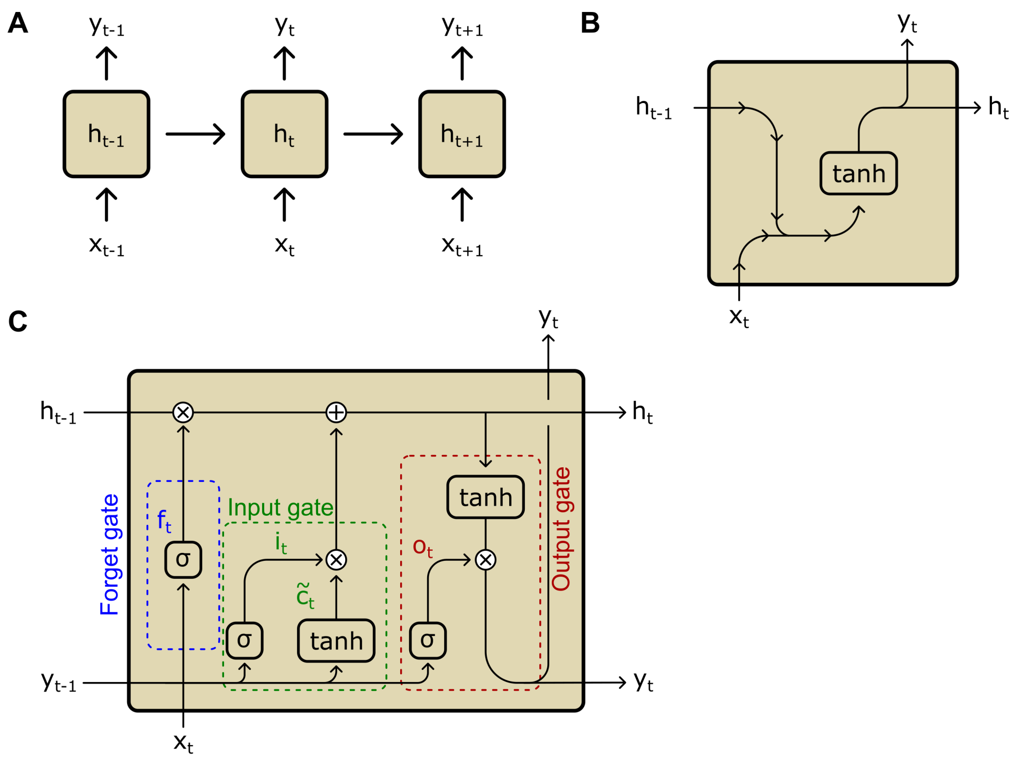

Figure S1. **A)** A recurrent neural network (RNN) is trained along a sequence of time points t. Based on input (x_t) and output data (y_t), a hidden state (h_t) is continuously updated using context units. **B)** A simple context unit in early RNNs, wherein the input from the previous time point of the hidden state (h_{t-1}) is combined with x_t and a $\tanh$ activation function to calculate y_t and update h_t. **C)** A Long Short-Term Memory (LSTM) cell is a more complex unit used to maintain h_t and determine y_t. Both sigmoidal (σ) and $\tanh$ activation functions are used within forget, input, and output gates, which determine which data is removed, added, and output from the cell. Figure inspired by existing work of Olah (2015).

$$i_t = \sigma(W_i[y_{t-1}, x_t] + b_i) \tag{S9}$$

$$\tilde{c}_t = \tanh(W_c[y_{t-1}, x_t] + b_c) \tag{S10}$$

Together, Equations S8 - S10 are combined to update h_t, where $*$ denotes pointwise multiplication (Equation S11).

$$h_t = f_t * h_{t-1} + i_t * \tilde{c}_t \tag{S11}$$

Finally, the output is determined by the output gates using x_t and y_{t-1} (Equation S12) and h_t (Equation S13).

$$o_t = \sigma(W_o[y_{t-1}, x_t] + b_o) \tag{S12}$$

$$y_t = o_t * \tanh(h_t) \tag{S13}$$

2.3 Transformers

For text data, tokenization is relatively straightforward, but for images, the ViT framework relies on patching, a process to divide a larger image into N smaller fixed-sized patches. These image patches are then linearly projected and embedded alongside their positions to encode the relative positions of each patch. These embedded patch and position data represent and thus are token representations of the original image data $\mathbf{T} = [t_i, ..., t_N]$. These tokens are then passed to attention models, where 'query' $\mathbf{q}$, 'key' $\mathbf{k}$, and 'value' $\mathbf{v}$ vectors for each token are calculated by their respective projection matrices (W_q, W_k, W_v) (Equation S14). These vectors are then compiled into larger matrices $\mathbf{Q}, \mathbf{K}, \mathbf{V}$ respectively (Equation S14).

$$\begin{aligned}
\mathbf{Q} &= \begin{bmatrix} \mathbf{q}_1^\top \\ \vdots \\ \mathbf{q}_N^\top \end{bmatrix} = \begin{bmatrix} (W_q t_1)^\top \\ \vdots \\ (W_q t_N)^\top \end{bmatrix} = \mathbf{T}\mathbf{W}_q^\top \\
\mathbf{K} &= \begin{bmatrix} \mathbf{k}_1^\top \\ \vdots \\ \mathbf{k}_N^\top \end{bmatrix} = \begin{bmatrix} (W_k t_1)^\top \\ \vdots \\ (W_k t_N)^\top \end{bmatrix} = \mathbf{T}\mathbf{W}_k^\top \\
\mathbf{V} &= \begin{bmatrix} \mathbf{v}_1^\top \\ \vdots \\ \mathbf{v}_N^\top \end{bmatrix} = \begin{bmatrix} (W_v t_1)^\top \\ \vdots \\ (W_v t_N)^\top \end{bmatrix} = \mathbf{T}\mathbf{W}_v^\top
\end{aligned} \tag{S14}$$

These vectors are then used to calculate an attention score which represents the saliency of the tokens $\mathbf{T}_{out}$ in an architecture referred to as self-attention (Equation S15), where $\sqrt{m}$ is a scaling factor based on the dimensionality of $\mathbf{K}$ to ensure stability during training.

$$\mathbf{T}_{out} = \text{softmax}\left(\frac{\mathbf{Q}\mathbf{K}^\top}{\sqrt{m}}\right)\mathbf{V} \tag{S15}$$

However, self-attention is relatively limited and, instead, multi-head self-attention (MHSA) is able to better capture map saliency and capture context. Essentially, MHSA has n parallel self-attention layers, each with their own learned projection matrices $\mathbf{W}_{q,i}, \mathbf{W}_{k,i}, \mathbf{W}_{v,i}$. Outputs from each layer are concatenated and projected using a learned output projection matrix $\mathbf{W}_o$ (Equation S16).

$$\begin{aligned}
\mathbf{T}_{out,multi} &= \text{concat}(\mathbf{T}_{out,1}, ..., \mathbf{T}_{out,n})\mathbf{W}_o \\
\text{where} \quad \mathbf{T}_{out,i} &= \text{softmax}\left(\frac{\mathbf{Q}_i\mathbf{K}_i^\top}{\sqrt{m}}\right)\mathbf{V}_i \\
\text{and} \quad \mathbf{Q}_i &= \begin{bmatrix} \mathbf{q}_{1,i}^\top \\ \vdots \\ \mathbf{q}_{N,i}^\top \end{bmatrix} = \begin{bmatrix} (W_{q,i}\, t_1)^\top \\ \vdots \\ (W_{q,i}\, t_N)^\top \end{bmatrix} = \mathbf{T}\mathbf{W}_{q,i}^\top \\
\mathbf{K}_i &= \begin{bmatrix} \mathbf{k}_{1,i}^\top \\ \vdots \\ \mathbf{k}_{N,i}^\top \end{bmatrix} = \begin{bmatrix} (W_{k,i}\, t_1)^\top \\ \vdots \\ (W_{k,i}\, t_N)^\top \end{bmatrix} = \mathbf{T}\mathbf{W}_{k,i}^\top \\
\mathbf{V}_i &= \begin{bmatrix} \mathbf{v}_{1,i}^\top \\ \vdots \\ \mathbf{v}_{N,i}^\top \end{bmatrix} = \begin{bmatrix} (W_{v,i}\, t_1)^\top \\ \vdots \\ (W_{v,i}\, t_N)^\top \end{bmatrix} = \mathbf{T}\mathbf{W}_{v,i}^\top
\end{aligned} \tag{S16}$$

REFERENCES

Miller MI, Trouvé A, Younes L. Geodesic Shooting for Computational Anatomy. *Journal of Mathematical Imaging and Vision* **24** (2006) 209–228. doi:10.1007/s10851-005-3624-0.

Vaillant M, Miller M, Younes L, Trouvé A. Statistics on diffeomorphisms via tangent space representations. *NeuroImage* **23** (2004) S161–S169. doi:10.1016/j.neuroimage.2004.07.023.

Miller MI, Trouvé A, Younes L. On the Metrics and Euler-Lagrange Equations of Computational Anatomy. *Annual Review of Biomedical Engineering* **4** (2002) 375–405. doi:10.1146/annurev.bioeng.4.092101.125733.

Durrleman S, Prastawa M, Charon N, Korenberg JR, Joshi S, Gerig G, et al. Morphometry of anatomical shape complexes with dense deformations and sparse parameters. *NeuroImage* **101** (2014) 35–49. doi:10.1016/j.neuroimage.2014.06.043.

Khare N, Thakur PS, Khanna P, Ojha A. *Analysis of Loss Functions for Image Reconstruction Using Convolutional Autoencoder* (Springer International Publishing) (2022), 338–349. doi:10.1007/978-3-031-11349-9_30.

Ehrhardt J, Wilms M. *Autoencoders and variational autoencoders in medical image analysis* (Elsevier) (2022), 129–162. doi:10.1016/b978-0-12-824349-7.00015-3.

Arpit D, Zhou Y, Ngo H, Govindaraju V. Why Regularized Auto-Encoders learn Sparse Representation? Balcan MF, Weinberger KQ, editors, *Proceedings of The 33rd International Conference on Machine Learning* (New York, New York, USA: PMLR) (2016), *Proceedings of Machine Learning Research*, vol. 48, 136–144.

Lipton ZC, Berkowitz J, Elkan C. A Critical Review of Recurrent Neural Networks for Sequence Learning (2015). doi:10.48550/ARXIV.1506.00019.

Salehinejad H, Sankar S, Barfett J, Colak E, Valaee S. Recent Advances in Recurrent Neural Networks (2018). doi:10.48550/ARXIV.1801.01078.

Staudemeyer RC, Morris ER. Understanding LSTM – a tutorial into Long Short-Term Memory Recurrent Neural Networks (2019). doi:10.48550/ARXIV.1909.09586.

Hochreiter S, Schmidhuber J. Long Short-Term Memory. *Neural Computation* **9** (1997) 1735–1780. doi:10.1162/neco.1997.9.8.1735.

Yu Y, Si X, Hu C, Zhang J. A Review of Recurrent Neural Networks: LSTM Cells and Network Architectures. *Neural Computation* **31** (2019) 1235–1270. doi:10.1162/neco_a_01199.

Chung J, Gulcehre C, Cho K, Bengio Y. Empirical evaluation of gated recurrent neural networks on sequence modeling (2014). doi:10.48550/ARXIV.1412.3555.

Olah C. Understanding lstm networks (2015).
